# Tomato *Sl3-MMP*, a member of the Matrix metalloproteinase family, is required for disease resistance against *Botrytis cinerea* and *Pseudomonas syringae* pv. *tomato* DC3000

**DOI:** 10.1186/s12870-015-0536-z

**Published:** 2015-06-14

**Authors:** Dayong Li, Huijuan Zhang, Qiuming Song, Lu Wang, Shixia Liu, Yongbo Hong, Lei Huang, Fengming Song

**Affiliations:** National Key Laboratory for Rice Biology, Institute of Biotechnology, Zhejiang University, Hangzhou, Zhejiang 310058 China

**Keywords:** Tomato (*Solanum lycopersicum*), Matrix metalloproteinases, *Botrytis cinerea*, *Pseudomonas syringae* pv. *tomato* DC3000, Disease resistance, Proteolysis

## Abstract

**Background:**

Matrix metalloproteinases (MMPs) are a family of zinc-dependent endopeptidases. MMPs have been characterized in detail in mammals and shown to play key roles in many physiological and pathological processes. Although MMPs in some plant species have been identified, the function of MMPs in biotic stress responses remains elusive.

**Results:**

A total of five *MMP* genes were identified in tomato genome. qRT-PCR analysis revealed that expression of *Sl-MMP* genes was induced with distinct patterns by infection of *Botrytis cinerea* and *Pseudomonas syringae* pv. *tomato (Pst)* DC3000 and by treatment with defense-related hormones such as salicylic acid, jasmonic acid and ethylene precursor 1-amino cyclopropane-1-carboxylic acid. Virus-induced gene silencing (VIGS)-based knockdown of individual *Sl-MMPs* and disease assays indicated that silencing of *Sl3-MMP* resulted in reduced resistance to *B. cinerea* and *Pst* DC3000, whereas silencing of other four *Sl-MMPs* did not affect the disease resistance against these two pathogens. The *Sl3-MMP*-silenced tomato plants responded with increased accumulation of reactive oxygen species and alerted expression of defense genes after infection of *B. cinerea*. Transient expression of *Sl3-MMP* in leaves of *Nicotiana benthamiana* led to an enhanced resistance to *B. cinerea* and upregulated expression of defense-related genes. Biochemical assays revealed that the recombinant mature Sl3-MMP protein had proteolytic activities *in vitro* with distinct preferences for specificity of cleavage sites. The Sl3-MMP protein was targeted onto the plasma membrane of plant cells when transiently expressed in onion epidermal cells.

**Conclusion:**

VIGS-based knockdown of *Sl3-MMP* expression in tomato and gain-of-function transient expression of *Sl3-MMP* in *N. benthamiana* demonstrate that *Sl3-MMP* functions as a positive regulator of defense response against *B. cinerea* and *Pst* DC3000.

**Electronic supplementary material:**

The online version of this article (doi:10.1186/s12870-015-0536-z) contains supplementary material, which is available to authorized users.

## Background

Proteases play key roles in the regulation of a variety of biological processes [1]. Matrix metalloproteinases (MMPs) are a family of zinc- and calcium-dependent proteases belonging to the metzincin clan of metalloendopeptidases, EC subclass 3.4.24, MA (M) clan according to the MEROPS database [2, 3]. The MMP family is characterized by the presence of a highly conserved catalytic domain containing an HEXXHXXGXX(H/D) zinc-binding sequence followed by a conserved methionine that forms a tight 1,4-β turn called Met-turn [4]. Members of this family have mainly been studied in mammals, but have also been found in simpler animals and plants [5]. In human, 23 MMP genes have been identified to encode proteins with similar structure, e.g., an N-terminal signal peptide for the secretory pathway, a prodomain that regulates the latency of the enzyme and a catalytic domain with the active zinc-binding site [6]. In addition, most of the human MMP proteins contain a C-terminal hemopexin (HPX)-like domain, which is believed to be important in regulating the activity and specificity of the catalytic domain [7, 8]. It has been shown that human MMPs play key roles in many physiological and pathological processes [9, 10].

Members of the MMP family have been identified in higher plants, but only few of them have been studied to date [2]. Similar to the human MMPs, the predicted primary structures of plant MMPs contain a signal peptide, a prodomain with the cysteine-switch motif and a catalytic domain containing the active zinc-binding sequence and structural zinc- and calcium-binding site followed by the conserved Met-turn [11–13]. Activation of MMPs requires physical delocalization of the prodomain from the catalytic site by proteolytic or nonproteolytic mechanisms [14]. It is believed that all plant MMPs are synthesized as inactive forms and are localized either in the plasma membrane or in the extracellular space. However, it was found that Arabidopsis At4-MMP contains a predicted non-cleavable N-terminal signal peptide and tobacco Nt1-MMP was inserted into the plasma membrane [15].

The biological function of MMP proteases in higher plants is largely unknown. Based on the expression patterns, it is proposed that the plant MMPs may be involved in remodeling of the extracellular matrix (ECM) during plant growth and development [2]. The first plant MMP was identified as an ethylenediaminetetraacetic acid (EDTA)-sensitive Azocoll-degrading enzyme in soybean [16]. In cucumber, Cs1-MMP was found to be associated with senescence and cell death in cotyledon development [17]. In Arabidopsis, *5 MMP* genes were identified and were found to be differentially expressed in roots, leaves, stems and flowers [15]. The *At2-MMP* mutant plants exhibited altered growth in association with late flowering and early senescence, supporting a physiological and developmental role for plant MMPs [18]. In *Medicago truncatula*, expression of *Mt1-MMP* was induced in young nodules, specifically in association with *Sinorhizobium meliloti* infection [13]. An *Mt1-MMP* RNAi mutant in *M. truncatula* showed nodules with enlarged infection threads and substantial increase in the number of bacterial colonies; whereas an ectopic overexpression of *Mt1-MMP* in roots led to a significant decrease in nodule number [13]. On the other hand, several lines of evidence also indicate that MMPs may be involved in biotic and abiotic stress responses in plants. In soybean, *Gm2-MMP* was isolated as a pathogen-induced gene [19]. Expression of *Gm2-MMP* was induced rapidly in compatible and incompatible interactions with pathogens, but not by salicylic acid (SA) and jasmonic acid (JA), two classical pathogen response signaling molecules [19]. In the tobacco suspension line BY-2, *Nt1-MMP* was expressed at low level but was induced immediately after treatment with *Pseudomonas syringae* [11]. In Arabidopsis, distinct expression patterns for each *MMP* in response to various abiotic and biotic stresses were described in the Genevestigator analysis [20]. *At3-MMP* showed significant changes in transcript levels under stress conditions, while other *MMPs* displayed minimal transcript changes [20]. The expression of *At2-MMP* is tightly controlled in a tissue-responsive way during stress conditions. *At2-MMP* in 4-week-old plants was induced in leaves by cadmium or methyl jasmonate and in roots by sodium chloride; however, cadmium inhibited the expression *of At2-MMP* in inflorescence and leaves of 10-week-old plants [18].

In the present study, we characterized the MMP family in tomato and performed functional analyses for their roles in disease resistance. A total of five MMP genes were identified in tomato and their expression was induced with distinct patterns in response to pathogen infection and treatments with defense-related hormones. Silencing of *Sl3-MMP* in tomato resulted in reduced resistance to *Botrytis cinerea* and *Pseudomonas syringae* pv. *tomato* (*Pst*) DC3000 whereas transient expression of *Sl3-MMP* in *Nicotiana benthamiana* led to an enhanced resistance to *B. cinerea*. Our data demonstrate that member of the MMP family may participate in the regulation of defense response in plants against pathogen infection.

## Results

### Identification of the Sl-MMP family in tomato

To identify members of the MMP family in tomato, HMM and Blastp searches using MMP proteins previously reported from Arabidopsis and other plant species as queries against the recently published tomato genome sequences (Release Version ITAG2.40) were performed. Five significant hits corresponding to non-redundant putative *Sl-MMP* genes were identified (Table 1). All five *Sl-MMPs* are intronless genes, which is consistent with structural features of genes for MMPs in Arabidopsis, soybean, cucumber and *Medicago truncatula* [13, 15, 17, 19, 21]. Full-length cDNAs for *Sl2-MMP* and *Sl3-MMP* were identified in the NCBI and SOL databases while no full-length cDNA was found for other three members (Table 1). We amplified and cloned all 5 *Sl-MMP* genes using gene-specific primers and confirmed by sequencing. These sequences were submitted to GenBank for deposition and presented in Additional file 1.

**Table 1:** Characterization of tomato Sl-MMP genes and proteins

Genes	Locus in SOL	Proteins in NCBI	Size (aa)	MW (kD)	*p*I	cDNAs in NCBI/SOL
*Sl1-MMP*	Solyc08g078550	XP_010325488	356	40.17	5.20	--
*Sl2-MMP*	Solyc04g005040	CCH68443	363	40.00	5.46	AK328733/AK327627/ SGN-U573509
*Sl3-MMP*	Solyc04g005050	NP_001266203	367	40.21	5.08	AK322919/ SGN-U573510
*Sl4-MMP*	Solyc05g006360	XP_010320628	357	40.50	6.00	--
*Sl5-MMP*	Solyc10g018750	XP_004248606	357	40.80	7.83	--

**Fig. 1:**
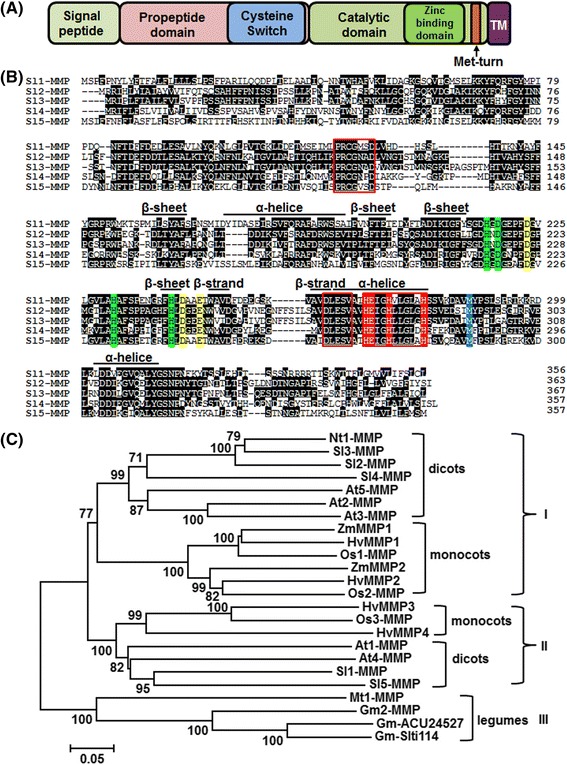
Sequence alignment and phylogenetic tree analysis of Sl-MMP with other plant MMP proteins. **a** Predicted domains of Sl-MMP proteins. TM, transmembrane domain. **b** Alignment of Sl-MMPs. Numbers on the right indicate amino acid positions of the Sl-MMP proteins. The cysteine switch motif, zinc-binding sequence and DLESV sequence are boxed in red. Secondary structure features such as β-strand, β-sheet and α-helix are indicated above the aligned sequences. The active site histidine and the catalytic glutamate residues are indicated in red. Ligands of the conserved structural zinc and calcium are colored in green and yellow, respectively. The hydrophobic base forming the methionine residue of the Met-turn is highlighted in blue. **c** Phylogenetic tree analysis of Sl-MMPs with other plant MMPs. Phylogenetic tree was constructed by Neighbor-joining method using MEGA program. Plant MMPs used and their GenBank accessions are as follows: *Arabidopsis thaliana* At1-MMP (NP_193397), At2-MMP (NP_177174), At3-MMP (NP_173824), At4-MMP (NP_182030), At5-MMP (NP_176205), *Glycine max* Gm2-MMP (AAL27029), Gm-ACU24527 (ACU24527), Gm-*Slti114* (ABW96008), *Hordeum vulgare* HvMMP1 (BAJ94792), HvMMP2 (BAJ93963), HvMMP3 (BAJ94176), HvMMP4 (BAJ90264), *Medicago truncatula* Mt1-MMP (CAA77093), *Nicotiana tabacum* NtMMP1 (ABF58910), *Solanum lycopersicum* Sl1-MMP (XP_010325488), Sl2-MMP (CCH68443), Sl3-MMP (NP_001266203), Sl4-MMP (XP_010320628), Sl5-MMP (XP_004248606), *Zea mays* ZmMMP1 (NP_001151749), ZmMMP2 (NP_001142095), *Oryza sativa* Os1-MMP (NP_001048075), Os2-MMP (NP_001057259), Os3-MMP (NP_001065361). Bootstrap values from 100 replicates are indicated at each node. Bar represents the number of amino acid differences per site

The Sl-MMP proteins are approximately 360 amino acids with molecular weight of ~40 kDa (Table 1). The Sl-MMP proteins shared conserved structural features, e.g., a signal sequence at N terminus, a propeptide domain, a catalytic domain, a transmembrane domain at C terminus (Fig. 1a). Characteristic motifs including a PRCGxxD motif, which is characteristic of the cysteine switch mechanism of activation [22], in the propeptide domain and a HExGHxxGxxH zinc-binding region and a conserved methionine residue in the Met-turn in the catalytic domain are present in the Sl-MMP proteins (Fig. 1b). In addition, each of Sl-MMPs contains an invariant DLESV motif on the N-terminal side of the zinc-binding region (Fig. 1b), which is thought to be a plant-specific motif with unknown function [15]. This motif is replaced by a distinct consensus sequence of NLFLV in human and insect MMPs [4, 23] but is not present in single-celled green algae MMPs [20, 24]. In term of secondary structure feature, Sl-MMPs have 2 β-strands, 4 β-sheets and 3 α-helices and they all contain three active site histidines and a catalytic glutamate residue in the zinc-binding region (Fig. 1a). Putative conserved structural ligands for binding zinc and calcium including 3 histidine (H), 2 aspartic acid (D) and 1 glutamic acid residues are present in Sl-MMPs (Fig. 1a). However, like MMPs from other plants, Sl-MMPs do not contain a C-terminal hemopexin domain, which is present in most human MMPs [8].

Phylogenetic tree analysis of Sl-MMPs with previously identified MMPs from other plant species clearly distinguished different groups, which had distinct features linked to plant species or specific functions. Groups I and II can be further separated into two subgroups, representing MMP branches from dicots and monocots. Sl2-MMP, Sl3-MMP and Sl4-MMP are assigned in Group I, which contain Nt1-MMP, At2-MMP and At3-MMP that are known to be pathogen-responsive [11, 18, 20]. Sl1-MMP and Sl5-MMP belong to Group II, whose members have been proposed to be involved in plant growth and development [20]. Group III only hosts MMPs from legumes such as *Glycine max* and *Medicago truncatula* and thus seem to be legume-specific.

### Expression of *Sl-MMPs* in response to pathogens and defense signaling-related hormones

**Fig. 2:**
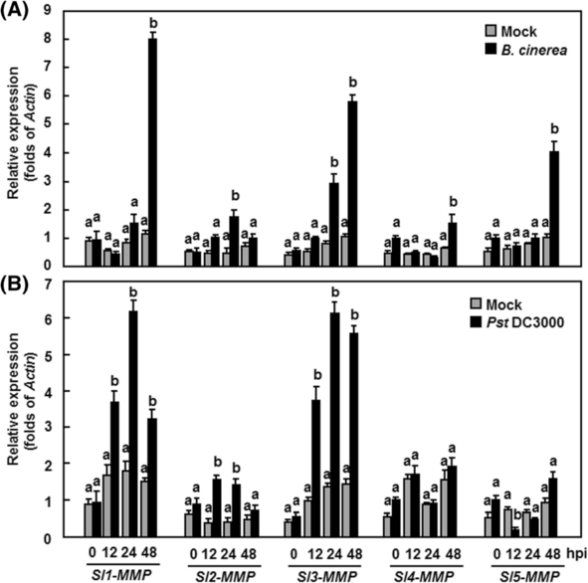
Expression patterns of *Sl-MMPs* in response to *B. cinerea* or *P. syringae* pv. *tomato* DC3000 treatment. Tomato plants were inoculated by spore suspension (2 × 10^5^ spores/ml) of *B. cinerea* or buffer solution as a mock-inoculation control (**a**) and by vacuum infiltration with *P. syringae* pv. *tomato* DC3000 (OD_600_ = 0.0002) or sterilized 10 mM MgCl_2_ solution as a mock-inoculation control (**b**). Leaf samples were collected at indicated time points and gene expression was analyzed by qRT-PCR. Relative expression levels were calculated by comparing with the corresponding values at 0 h (as a control) after inoculation and shown as folds of the actin transcript values. Data presented are the means ± SD from three independent experiments and different letters above the columns indicate significant differences at *p* < 0.05 level

To explore the possible involvement of *Sl-MMPs* in defense response against pathogen infection, we first analyzed the expression changes of *Sl-MMPs* after infection with *B. cinerea*. As shown in Fig. 2a, expression of the *Sl-MMP* genes were induced upon infection of *B. cinerea* but showed distinct expression patterns. Generally, the expression of *Sl1-MMP*, *Sl3-MMP*, *Sl4-MMP* and *Sl5-MMP* was significantly induced with peaks at 48 h whereas the expression of *Sl2-MMP* was induced significantly with peaks at 24 h after infection with *B. cinerea*, as compared with those in the mock-inoculated plants (Fig. 2a). Specifically, the expression levels of *Sl1-MMP*, *Sl3-MMP* and *Sl5-MMP* in *B. cinerea*-infected plants showed >5 folds of increases over those in the mock-inoculated plants at 48 h after inoculation (Fig. 2a). The expressions of *Sl1-MMP* and *Sl3-MMP* exhibited 4-5 folds of increases at 24 h after infection of *B. cinerea*. It was noted that the expression of *Sl1-MMP*, *Sl4-MMP* and *Sl5-MMP* was induced only at 48 h after infection of *B. cinerea* (Fig. 2a). These results indicate that the *Sl-MMP* genes respond with different dynamics and magnitude of expression after infection of *B. cinerea*.

**Fig. 3:**
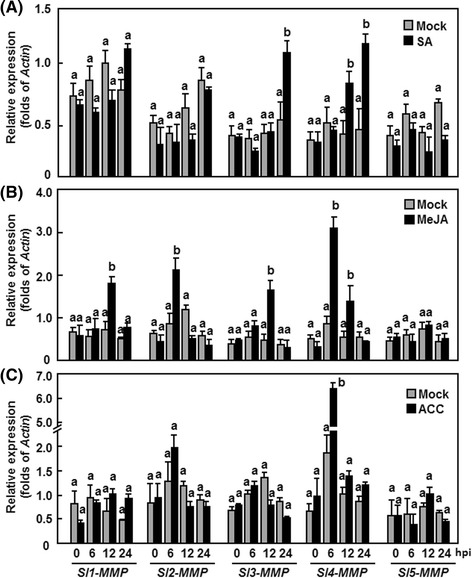
Expression patterns of *Sl-MMPs* in response to defense signaling hormones. Tomato plants were treated by foliar spraying of 1 mM SA (**a**), 100 μM MeJA (**b**), 100 μM ACC (**c**) or equal volume of solution as a control and leaf samples were collected at indicated time points. Gene expression was analyzed by qRT-PCR and relative expression levels were calculated by comparing with the corresponding values at 0 h (as a control) after treatment. Relative expression was shown as folds of the actin transcript values. Data presented are the means ± SD from three independent experiments and different letters above the columns indicate significant differences at *p* < 0.05 level

We next analyzed the expression changes of *Sl-MMPs* after infection with *Pst* DC3000. As shown in Fig. 3b, only *Sl1-MMP*, *Sl2-MMP* and *Sl3-MMP* were induced upon infection of *Pst* DC3000 and again showed different expression patterns. Generally, the expression of *Sl1-MMP* and *Sl3-MMP* was induced significantly with peaks at 24 h while the expression of *Sl2-MMP* was induced significantly with peaks at 12 h after infection with *Pst* DC3000, as compared with those in the mock-inoculated plants (Fig. 2b). Specifically, the expression of *Sl1-MMP* and *Sl3-MMP* in *Pst* DC3000-infected plants showed >5 folds of increases over those in the mock-inoculated plants at 24 h, whereas the expression of *Sl2-MMP* was induced significantly with peaks at 12 and 24 h after infection with *Pst* DC3000 (Fig. 2b). Interestingly, the expression of *Sl5-MMP* was down-regulated at 12 h (Fig. 2b). These results indicate that the expression of *Sl1-MMP*, *Sl2-MMP* and *Sl3-MMP* was induced by *Pst* DC3000.

**Fig. 4:**
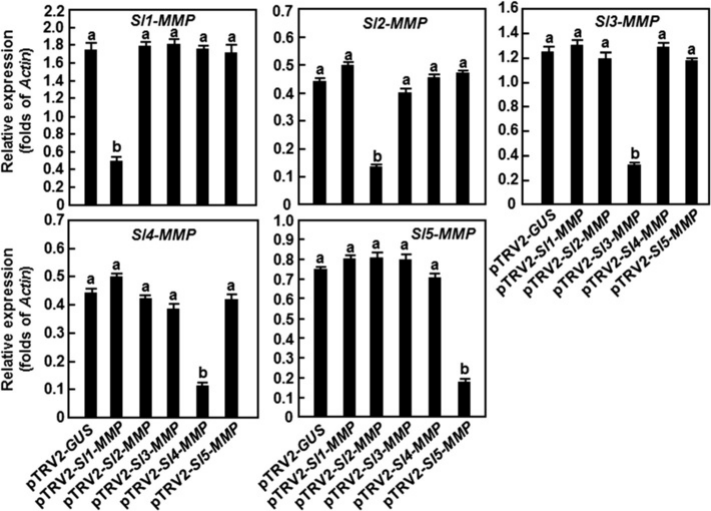
Silencing efficiency and specificity for target genes in silenced plants. Two-week-old tomato seedlings were infiltrated with agrobacteria carrying pTRV2-Sl-MMPs or pTRV2-GUS and leaf samples were collected at 4 weeks after agroinfiltration. Expression levels of each *Sl-MMP* genes in targeted and nontargeted *Sl-MMP*-silenced and non-silenced plants were analyzed by qRT-PCR and data obtained were normalized with actin transcript values. Data presented are the means ± SD from three independent experiments and different letters above the columns indicate significant differences at *p* < 0.05 level

We also examined the dynamics of *Sl-MMPs* expressions in tomato plants after treatments with SA, methyl jasmonate (MeJA) and 1-amino cyclopropane-1-carboxylic acid (ACC) [a precursor of ethylene (ET)], three defense signaling-related hormones. As shown in Fig. 4, different expression patterns for *Sl-MMPs* were observed in response to these defense signaling-related hormones. In SA-treated plants, expression of *Sl3-MMP* and *Sl4-MMP* was significantly increased by 2-3 folds over that in the control plants, while expressions of *Sl1-MMP*, *Sl2-MMP* and *Sl3-MMP* were not affected (Fig. 3a). In JA- or ACC-treated plants, expression of *Sl4-MMP* was strongly induced, reaching 3-4 folds of increased at 6 h after treatment (Fig. 3b and c). Besides *Sl4-MMP*, the expression of *Sl1-MMP*, *Sl2-MMP* and *Sl3-MMP* was also induced by JA, showing an increase of 2-3 folds at 12 h (Fig. 3b). Except *Sl4-MMP*, expression of other four *Sl-MMPs* was not affected by ACC (Fig. 3c). Interestingly, the expression of *Sl5-MMP* was not affected by both JA and ACC during the experimental period (Fig. 3b and c). These data indicate that the tomato *Sl-MMPs* respond with different expression patterns to SA, JA and ET, three well-known defense signaling-related hormones.

### Silencing of *Sl3-MMP* resulted in reduced resistance to *B. cinerea* and *Pst* DC3000

To examine the possible involvement of *Sl-MMPs* in disease resistance, we performed functional analyses by virus-induced gene silencing (VIGS) approach through comparing the disease phenotype between individual *Sl-MMP*-silenced plants and non-silenced control plants. For this purpose, specific fragment for each *Sl-MMP* gene (Additional file 2) was chosen to generate VIGS construct and standard VIGS procedure with a *phytoene desaturase* (*PDS*) construct as an indicative for VIGS efficiency of each experiment was performed on 2-week-old plants [25, 26]. Under our experiment conditions, >90 % of the pTRV2-*PDS*-infiltrated plants showed bleaching phenotype (data not shown). The silencing efficiency and specificity for each *Sl-MMP* gene was examined by qRT-PCR analyzing the transcript level of the target *Sl-MMP* gene and other four *Sl-MMP* genes in the pTRV2-target *Sl-MMP*-infiltrated plants. When compared with those in the pTRV2-GUS-infiltrated plants, the transcript level of the target *Sl-MMP* gene was significantly reduced whereas the transcript levels of the other *Sl-MMP* genes were comparable in the pTRV2-target *Sl-MMP*-silenced plants (Fig. 4). Overall, the silencing efficiency for a target *Sl-MMP* gene was approximately 70 % (Fig. 4). The efficiencies and specificity of silencing for each individual *Sl-MMP* gene were satisfied for further experiments and all the subsequent experiments were performed only on those pTRV2-Sl-MMPs-infiltrated plants with high levels of silencing efficiency (>70 %).

**Fig. 5:**
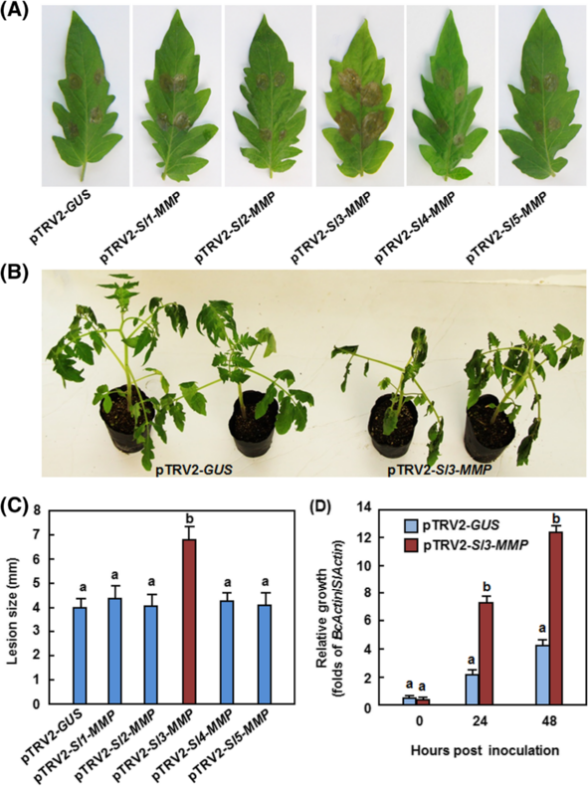
Silencing of *Sl3-MMP* resulted in reduced resistance to *B. cinerea*. Two-week-old seedlings were infiltrated with agrobacteria carrying pTRV2-Sl-MMP or pTRV2-GUS and were inoculated at 4 weeks after VIGS infiltration by dropping spore suspension (1 × 10^5^ spores/mL) on detached leaves or foliar spraying with spore suspension (2 × 10^5^ spores/mL) onto leaves of whole plants. **a** and **b** Disease phenotype and lesion sizes in leaves of the pTRV2-Sl-MMPs- and pTRV2-GUS-infiltrated plants in detached leaf inoculation assays. Lesion sizes were measured at 3 days after inoculation on a minimum of 20 leaves in each experiment. **c** and **d** Disease phenotype on and fungal growth in the pTRV2-Sl3-MMP- and pTRV2-GUS-infiltrated plants in whole plant inoculation assays. Fungal growth *in planta* was estimated by analyzing the transcript levels of *BcActin* gene by qRT-PCR using *SlActin* as an internal control at the indicated time points after inoculation. Data presented in **b** and **d** are the means ± SD from three independent experiments and different letters above the columns indicate significant differences at *p* < 0.05 level

We first examined the possible involvement of *Sl-MMPs* in resistance against *B. cinerea* by challenging the pTRV2-Sl-MMPs-infiltrated plants with spore suspension of *B. cinerea* and comparing the disease severity and in *planta* fungal growth with those in pTRV-GUS-infiltrated non-silenced plants. In our detached leaf assays, *B. cinerea*-caused lesions on detached leaves from the pTRV2-Sl1-MMP-, pTRV2-Sl2-MMP-, pTRV2-Sl4-MMP- and pTRV2-Sl5-MMP-infiltrated plants were similar to those on the detached leaves from pTRV2-GUS-infilrtratd plants (Fig. 5a), suggesting that *Sl1-MMP*, *Sl2-MMP*, *Sl4-MMP* and *Sl51-MMP* may not be involved in resistance against *B. cinerea*. However, *B. cinerea*-caused lesions on detached leaves from the pTRV2-Sl3-MMP-infiltrated plans were significantly larger and developed faster, merging into large necrotic areas, as compared with those on leaves from the pTRV2-GUS-infiltrated plants (Fig. 5a), at 3 days after inoculation (dpi), showing an approximately 60 % of increase in lesion size over those on leaves from the pTRV2-GUS-infiltrated control plants (Fig. 5c). We further analyzed and compared the disease severity and *in planta* fungal growth in the pTRV2-Sl3-MMP- and pTRV2-GUS-infiltrated plants after inoculation by foliar spraying with spore suspension of *B. cinerea* in whole plant inoculation experiments. As shown in Fig. 5b, the pTRV2-GUS-infiltrated control plants displayed slight disease symptoms, whereas the pTRV2-Sl3-MMP-infiltrated plants showed severe diseases symptoms, showing large necrotic areas and maceration or wilting of full leaves at 5 dpi. Analysis of the transcript for the *B. cinerea* actin gene *BcActin* revealed that growth of *B. cinerea* in leaf tissues of the pTRV2-Sl3-MMP-infiltrated plants had 3 times higher than those in the pTRV2-GUS-infiltrated control plants at 24 and 48 h after inoculation (Fig. 5d). These data indicate that silencing of the *Sl3-MMP* resulted in reduced resistance to *B. cinerea*, demonstrating the requirement of *Sl3-MMP* for resistance to *B. cinerea*.

**Fig. 6:**
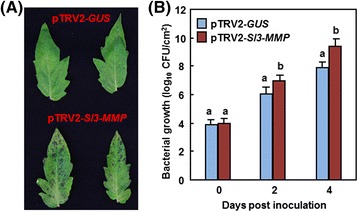
Silencing of *Sl3-MMP* resulted in reduced resistance to *P. syringae* pv. *tomato* DC3000. Two-week-old seedlings were infiltrated with agrobacteria carrying pTRV2-Sl3-MMP or pTRV2-GUS and were inoculated by infiltration with *Pst* DC3000 4 weeks after VIGS infiltration. **a** Representative disease phenotype. **b** Bacterial population. Data presented in **b** are the means ± SD from three independent experiments and different letters above the columns indicate significant differences at *p* < 0.05 level

As mentioned above that silencing of *Sl3-MMP* resulted in a clear phenotype in change of disease resistance to *B. cinerea*, we subsequently focused our efforts on the functions in resistance to other diseases, mechanism and biochemical activity of Sl3-MMP. We examined whether *Sl3-MMP* is also involved in resistance against *Pst* DC3000, which is a hemibiotrophic bacterial pathogen that has different infection style from that of *B. cinerea*. In our experiments, necrotic lesions were observed in the inoculated leaves of the pTRV2-Sl3-MMP- and pTRV2-GUS- infiltrated plants; however, the lesions on leaves of the pTRV2-Sl3-MMP-infiltrated plants were larger and denser than those in the pTRV2-GUS-infiltrated plants (Fig. 6a). At 2 and 4 dpi, the bacterial population in the inoculated leaves of the pTRV2-Sl3-MMP-infiltrated plants showed approximately 10 and 25 folds higher over those in the pTRV2-GUS-infiltrated plants, respectively (Fig. 6b). These results indicate that silencing of *Sl3-MMP* resulted in reduced resistance to *Pst* DC3000, implying the requirement of *Sl3-MMP* for resistance against *Pst* DC3000.

### Silencing of *Sl3-MMP* attenuated defense response against *B. cinerea*

**Fig. 7:**
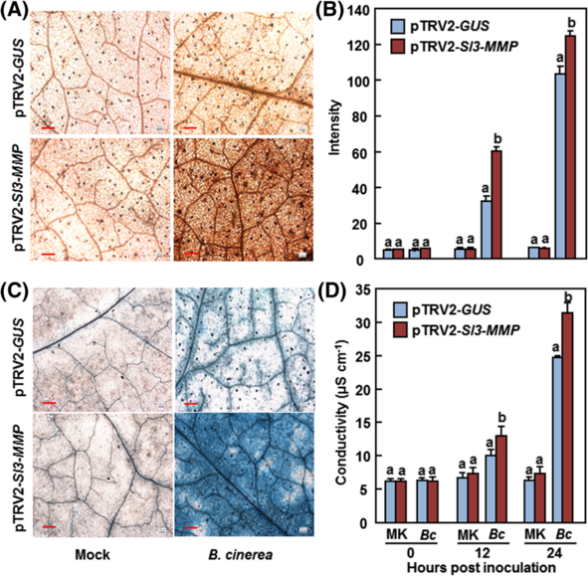
Increased accumulation of H_2_O_2_ and cell death in Sl3-MMP-silenced plants after infection with *B. cinerea*. Two-week-old seedlings were infiltrated with agrobacteria carrying pTRV2-Sl3-MMP or pTRV2-GUS and were inoculated with spore suspension of *B. cinerea* or with buffer solution as a mock-inoculation control at 4 weeks after VIGS infiltration. Leaves from six individual plants were collected at 24 h after inoculation and used for analysis of cell death and H_2_O_2_ accumulation. **a** and **b** Accumulation of H_2_O_2_ by DAB staining and quantification method respectively. **c** Cell death detected by trypan blue staining. **d** Electrolyte leakage. Data presented in **b** and **d** are the means ± SD from three independent experiments and different letters above the columns indicate significant differences at *p* < 0.05 level

To elucidate the possible mechanism involved in the reduced resistance in *Sl3-MMP*-silenced plants, we analyzed and compared the accumulation of reactive oxygen species (ROS)_,_ cell-death response and expression of defense genes before and after infection with *B. cinerea* between the *Sl3-MMP*-silenced plants and the control plants. No difference in accumulation of H_2_O_2_, as detected by 3, 3-diaminobenzidine (DAB) staining, was observed in leaves of pTRV2-Sl3-MMP- and pTRV2-GUS-infiltrated plants without infection of *B. cinerea* (Fig. 7a), indicating that silencing of *Sl3-MMP* itself did not affect the generation and accumulation of H_2_O_2_ in tomato plants. After infection with *B. cinerea*, significant accumulation of H_2_O_2_, shown as brown precipitates in leaves, was detected in leaves of pTRV2-Sl3-MMP- and pTRV2-GUS-infiltrated plants (Fig. 7a). However, the leaves from pTRV2-Sl3-MMP-infiltrated plants showed consistent increase in intensity of the stained areas (Fig. 7a), showing increases of 85 % at 12 h and 24 % 24 h, when compared with those in pTRV2-GUS-infiltrated plants after infection of *B. cinerea* (Fig. 7b). On the other hand, levels of cell death, as detected by Trypan blue staining, and electrolyte leakage, as estimated by ion conductivity, were comparable in leaves of pTRV2-Sl3-MMP- and pTRV2-GUS-infiltrated plants without infection of *B. cinerea* but significantly increased after infection with *B. cinerea* (Fig. 7c and d). Notably, the levels of cell death and electrolyte leakage in leaves of pTRV2-Sl3-MMP-infiltrated plants were significantly higher than those in leaves of pTRV2-GUS-infiltrated plants after infection of *B. cinerea* (Fig. 7c), leading to 38 % increases for electrolyte leakage at 24 h after infection (Fig. 7d). These data indicate that silencing of *Sl3-MMP* resulted in increased ROS accumulation of H_2_O_2_ and excessive cell death in pTRV2-Sl3-MMP-infiltrated plants upon infection of *B. cinerea*.

**Fig. 8:**
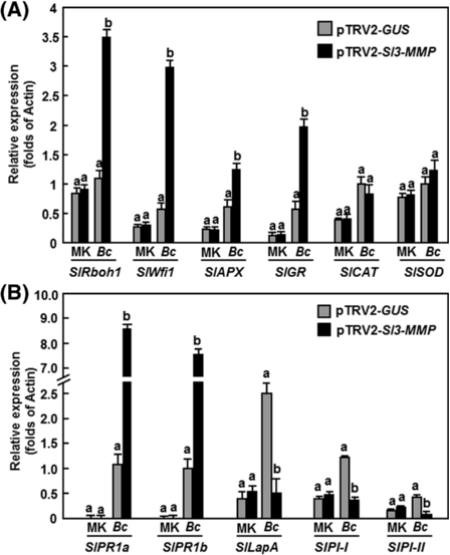
Silencing of *Sl3-MMP* affected the expression of ROS generation- and scavenging-related genes and defense-mediated genes after infection of *B. cinerea*. Two-week-old seedlings were infiltrated with agrobacteria carrying pTRV2-Sl3-MMP or pTRV2-GUS and were inoculated with spore suspension of *B. cinerea* or with buffer as a mock-inoculation control at 4 weeks after VIGS infiltration. Leaves from six individual plants were collected at 24 h after inoculation and used for analysis of gene expression. **a** Expression of ROS generation- and scavenging-related genes. **b** Expression of defense-related genes. Relative expression levels were shown as folds of the actin transcript values. Data presented are the means ± SD from three independent experiments and different letters above the columns indicate significant differences at *p* < 0.05 level. MK, mock-inoculated control; Bc, *B. cinerea*-inoculated treatment

To explore the possible mechanism for the increased accumulation of H_2_O_2_ in the Sl3-MMP-silenced plants, we analyzed and compared the expression of genes encoding for NADPH oxidases, glutathione reductase (GR), catalases (CAT), superoxide dismutases (SOD) and ascorbate peroxidases (APX) in the pTRV2-Sl3-MMP-infiltrated plants. As shown in Fig. 8a, no significant difference in the expression levels of these selected genes was observed between the pTRV2-GUS-infiltrated and pTRV2-Sl3-MMP-infiltrated plants without infection of *B. cinerea*. By contrast, the expression levels of *Rboh1* and *Wfi1*, two genes for NADPH oxidases, in the pTRV2-Sl3-MMP-infiltrated plants were significantly elevated upon *Botrytis* infection, showing ~5-fold increases over those in the pTRV2-GUS-infiltrated plants. Similarly, the expression levels of *APX* and *GR* in the pTRV2-Sl3-MMP-infiltrated plants were also increased as compared with those in the pTRV2-GUS-infiltrated plants (Fig. 8a). By contrast, no significant difference was observed in the expression levels of *CAT* and *SOD* between the pTRV2-Sl3-MMP-infiltrated plants and the pTRV2-GUS-infiltrated plants (Fig. 8a). These results indicate that the increased ROS accumulation in the Sl3-MMP-silenced plants might be due to an increased ROS generating ability resulted from the high level of expression of the NADPH oxidases.

We next analyzed the expression changes of defense-related genes regulated by the JA/ET- and SA-mediated signaling pathways, respectively, to explore the possible molecular mechanism associated with the reduced disease resistance in *Sl3-MMP*-silenced plants. No significant difference in expression of *SlPR1a* and *SlPR1b*, known to be regulated by the SA-mediated signaling pathway [27], and *SlLapA*, *SlPI-I* and *SlPI-II*, known to be regulated by the JA/ET-mediated signaling pathway [27], was observed in mock-inoculated pTRV2-Sl3-MMP- or pTRV2-GUS-infiltrated plants (Fig. 8b), indicating that silencing of *Sl3-MMP* did not affect the expression of defense-related genes in tomato plants. However, the expression of these defense-related genes exhibited different patterns in pTRV2-Sl3-MMP- or pTRV2-GUS-infiltrated plants after infection of *B. cinerea* (Fig. 8b). The expression of *SlPR1a* and *SlPR1b* in the pTRV2-Sl3-MMP- and pTRV2-GUS-infiltrated plants was significantly upregulated after infection of *B. cinerea*, but the levels in pTRV2-Sl3-MMP-infiltrated plants showed 7-8 folds higher than those in the pTRV2-GUS-infiltrated plants (Fig. 8b). After infection with *B. cinerea*, the expression levels of *SlLapA*, *SlPI-I* and *SlPI-II* in pTRV2-GUS-infiltrated plants were significantly increased, whereas the levels in pTRV2-Sl3-MMP-infiltrated plants remained unchanged, comparable to those in the mock-inoculated plants but showing 3-5 folds of decreases as compared with those in *B. cinerea*-infected pTRV2-GUS-infiltrated plants (Fig. 8b). These data demonstrate that silencing of *Sl3-MMP* attenuated the defense response in tomato upon infection of *B. cinerea* through affecting the expression of defense-related genes that are regulated by the JA/ET-mediated signaling pathway.

### Transient expression of *Sl3-MMP* in *Nicotiana benthamiana* led to increased resistance against *B. cinerea*

**Fig. 9:**
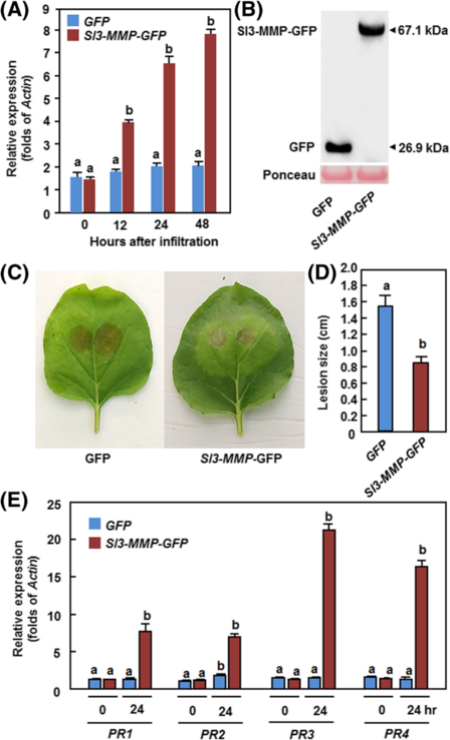
Transient expression of *Sl3-MMP* in *N. benthamiana* conferred an increased resistance to *B. cinerea*. **a** Expression of *Sl3-MMP*. Agrobacteria carrying pFGC-Sl3-MMP or pFGC-eGFP were infiltrated into leaves of *N. benthamiana* and expression of *Sl3-MMP* was analyzed by qRT-PCR. Relative expression levels were calculated by comparing with the corresponding values at 0 h (as a control) after infiltration. **b** Immunoblot analysis of Sl3-MMP-GFP fusion proteins in *N. benthamiana* leaves at 48 h after agroinfiltration. A GFP-specific antibody was used for detection of GFP-fusion protein. Equal loading of total proteins was examined by Ponceau staining. **c** and **d** Disease symptom and lesion size. **e** Expression of defense-related genes. Opposite part of the leaves infiltrated with Sl3-MMP*-*GFP or pFGC-eGFP was inoculated by dropping spore suspension (2 × 10^5^ spores/mL) of *B. cinerea* and lesion sizes were measured at 5 days after inoculation. Data presented in **d** are the means ± SD from a minimum of 60 lesions. Data presented in **a** and **e** are the means ± SD from three independent experiments and different letters above the columns indicate significant differences at *p* < 0.05 level

To further confirm the function of *Sl3-MMP* in disease resistance, we examined whether overexpression of *Sl3-MMP* could confer an increased resistance to *B. cinerea*. In our qRT-PCR experiments, transcripts of putative *N. benthamiana* homolog(s) of *Sl3-MMP* was detected using Sl3-MMP-specific primers in GFP-infiltrated plants, probably due to high level of sequence similarity/identity among *Sl3-MMP* and the homologous *MMP* genes in *N. benthamiana*. However, agroinfiltration did not significantly affect the transcript levels of endogenous *N. benthamiana* homologous genes in GFP-infiltrated plants (Fig. 9a). When transiently expressed in *N. benthamiana* leaves, high levels of *Sl3-MMP* expression, as estimated by the significant increases in the transcript levels of *Sl3-MMP* and the endogenous homologous genes in Sl3-MMP-infiltrated plants over the levels of the endogenous homologous genes in GFP-infiltrated plants, and the Sl3-MMP-GFP (a fusion of Sl3-MMP with GFP) fusion protein were detected during a period of 48 h after infiltration (Fig. 9a and b). In disease assays, the lesions on leaves from Sl3-MMP-infiltrated *N. benthamiana* plants were significantly smaller than that in GFP-infiltrated control plants (Fig. 9c), leading to approximately 40 % of reduction in lesion size at 5 days after inoculation (Fig. 9d). To examine whether an increased defense response was linked to the enhanced resistance resulted from the transient expression of *Sl3-MMP*, we analyzed and compared the expression of some selected defense-related genes in leaves of the GFP- and Sl3-MMP-infiltrated *N. benthamiana* plants. As shown in Fig. 9e, the expression of *PR1*, *PR2*, *PR3* and *PR4* in Sl3-MMP-infiltrated plants were significantly upregulated at 24 h after infiltration, showing 5-24 folds of increases over those in GFP-infiltrated plants, whereas no significant difference in the levels of these defense-related genes was observed between GFP-infiltrated plants at 0 h and 24 h and between GFP- and Sl3-MMP-infiltrated plants at 0 h after infiltration (Fig. 9e). These data demonstrate that transient expression of *Sl3-MMP* in *N. benthamiana* plants conferred an increased resistance against *B. cinerea* through an activated defense response resulted from the upregulated expression of defense-related genes.

### Proteolytic activity and subcellular localization of Sl3-MMP

**Fig. 10:**
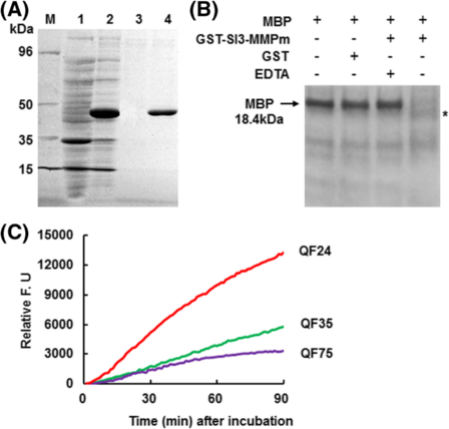
Mature Sl3-MMP protein had proteolytic activity with different cleavage site specificity. **a** Purification of recombinant Sl3-MMPm. M, marker for protein molecular weight; lane 1, total cell proteins from non-induced bacteria carrying pGEX-Sl3-MMPm; lane 2, total cell protein from induced bacteria carrying pGEX-Sl3-MMPm; lane 3, purified protein from non-induced bacteria carrying pGEX-Sl3-MMPm; lane 4, purified Sl3-MMP protein. **b** Proteolytic activity of Sl3-MMPm on MBP in the absence (-) or presence (+) of 1 mM EDTA. Reactions with different component combinations were incubated with 5 μg of MBP at 37 °C and the products were resolved by 16 % SDS-PAGE. Lane 1, a negative control containing MBP only; Lane 2, a negative control with GST tag; Lane 3, a reaction containing Sl3-MMPm and 1 mM EDTA; lane 4, a reaction containing Sl3-MMPm. * indicates the product degraded from MBP by Sl3-MMPm. **c** Cleavage site specificity of Sl3-MMPm on synthetic quenched peptide substrates. Enzymatic activity of Sl3-MMPm on synthetic quenched peptides QF24, QF35 and QF75 was measured as relative fluorescence in arbitrary units over 90 min at 1 min intervals. Values are the means of triplicate measurements

To delineate the biochemical activity of Sl3-MMP, we expressed the putative mature form of Sl3-MMP (Gly^155^-Ser^340^), in which the N-terminal propeptide domain as well as the C-terminal predicted transmembrane domains were deleted, in *E. coli*, and purified the recombinant mature Sl3-MMP protein (Sl3-MMPm) to homogeneity as examined on a SDS-PAGE gel (Fig. 10a). The activity of the recombinant Sl3-MMPm was examined for its ability to cleave a general protease substrate bovine myelin basic protein (MBP). As shown in Fig. 10b, MBP was present as a single band without any degradation in absence of Sl3-MMPm and the GST tag alone did not cause degradation of MBP. However, significant degradation of MBP was detected in the presence of Sl3-MMPm, showing an additional band with small molecular weight (Fig. 10b). Meanwhile, addition of 1 mM EDTA, an inhibitor of MMP [2], in the Sl3-MMPm-MBP reaction completely abolished the degradation of MBP by Sl3-MMPm (Fig. 10b). These results demonstrate that the recombinant Sl3-MMPm had proteolytic activity on MBP. To determine the cleavage site specificity, we examined whether Sl3-MMPm was able to hydrolyze quenched fluorescent (QF) peptide substrates QF24, QF35 and QF75. As shown in Fig. 10c, the recombinant Sl3-MMPm cleaved the QF24 most efficiently, followed by QF 35 and then QF75, indicating a cleavage site specificity of Sl3-MMP towards different substrates.

**Fig. 11:**
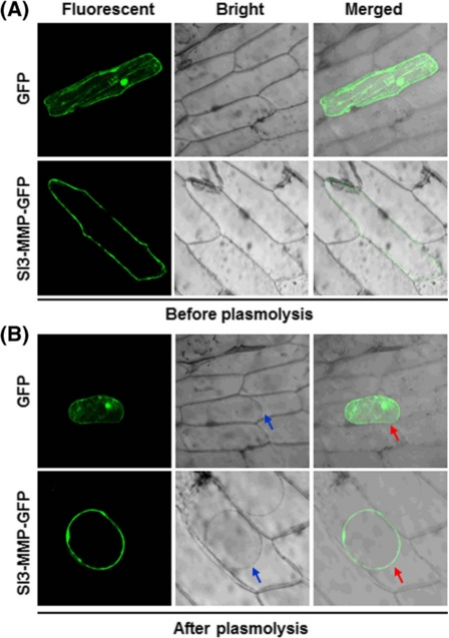
Sl3-MMP was targeted to plasma membrane in onion epidermal cells. Plasmids pFGC-Sl3-MMP and pFGC-eGFP alone were introduced into onion epidermal cells by particle bombardment and fluorescence was observed before (**a**) and after (**b**) plasmolysis. Plasmolysis was achieved by treating the epidermal cells with 0.8 M mannitol. Same cells were viewed simultaneously under fluorescent field for GFP and bright field for the intact cells. Merged images of confocal GFP fluorescence and bright field are also presented. Blue arrows indicate the cells undergoing plasmolysis and red arrows indicate the GFP signals on plasma membrane or in cells after plasmolysis

Subcellular localization of Sl3-MMP was examined by transient expression of a GFP-tagged Sl3-MMP construct in onion epidermal cells. To exclude the possibility that Sl3-MMP-GFP is secreted into the apoplast and not associated with the plasma membrane, the GFP-tagged Sl3-MMP construct was introduced into onion epidermal cells by particle bombardment and GFP was observed before and after plasmolysis, which was induced by rinsing the cells with 0.8 M mannitol solution for 10 min. The GFP protein alone accumulated in the cells without specific localization; however, GFP from the GFP-tagged Sl3-MMP protein was clearly localized on the plasma membrane before and after plasmolysis and no GFP was detected in the space between plasma membrane and cell wall (Fig. 11). These observations indicate that Sl3-MMP was targeted to plasma membrane of cells but not secreted into the apoplast.

## Discussion

MMPs are a family of zinc-dependent endopeptidases widely distributed in all organisms. However, only a few of plant MMPs have been studied for their biological functions so far [2]. In the present study, we characterized the tomato MMP family, analyzed the expression patterns of *Sl-MMP* genes in response to pathogen infection and treatments of defense-related signaling hormones, and performed VIGS- and transient expression-based functional analyses to explore the involvement of *Sl-MMPs* in disease resistance. Our results demonstrate that Sl3-MMP act as a positive regulator of defense response against *B. cinerea* and *Pst* DC3000 in tomato, providing new insights into the biological function of plant MMPs.

The significance of the proteases is generally related to their substrates and the physiological consequences of these actions. No physiological substrate has been identified for higher plant MMPs. However, activity of plant MMPs was detected *in vitro* on general animal protease substrates [11, 12, 15, 17, 19, 28, 29]. It was also found that recombinant mature MMPs, lacking the N-terminal propeptide domains showed higher proteolytic activity than the full-length MMPs [15, 19, 28]. In the present study, we demonstrated that Sl3-MMPm, lacking the N-terminal propeptide domain, had proteolytic activity toward MBP *in vitro* (Fig. 10b). Similar proteolytic activity on MBP was verified for At1-MMP, At2-MMP, At5-MMP [15, 30], Gm2-MMP [19] and Pta1-MMP [12]. However, proteolytic activities of plant MMPs on other substrates were also detected, e.g., Cs1-MMPand Nt1-MMP on gelatin [17, 28] and At3-MMP and Nt1-MMP on β-casein [11, 28, 30]. Therefore, it is likely that members of plant MMP family may have different substrates in plants and such substrate specificity may determine their biological functions. On the other hand, previous reports have shown that both human and plant MMPs differ in their cleavage site preferences [30]. Synthetic peptides, designed for vertebrate MMPs, have been used to characterize the proteolytic activity of SMEP1/Gm1-MMP [29], At1-MMP-At5-MMP [15, 30], Cs1-MMP [17] and Nt1-MMP [28]. Among these synthetic peptides, QF24 is a substrate for all MMPs [31], QF35 is the stromelysin substrate [32] and QF75 was designed to mimic the activation cleavage site in human MMP-2 and has an amino acid sequence different from the typical MMP cleavage motif [30]. In the present study, we found that Sl3-MMPm, lacking the N-terminal propeptide domain, cleaved the QF24 with the highest efficiency, followed by QF35 and QF75 (Fig. 10c). Thus, Sl3-MMP is able to efficiently cleave general MMP substrates. This is in agreement with the differences in the cleavage site specificities observed in Arabidopsis MMPs [30]. For example, At1-MMP was found to cleave efficiently QF24 and QF35 while only At5-MMP was found to be able to cleave QF75 [15, 30].

Most human MMPs are secreted into the extracellular matrix, but six membrane-type MMPs maintain contact with the cell surface through either transmembrane domains or glycosylphosphatidylinositol (GPI) anchors [33]. Like some plant MMPs such as GmMMP2, At2-MMP, At4-MMP and At5-MMP [20], Sl3-MMP contains a signal peptide (1-21 aa) at the N-terminus and a putative GPI-anchor modification site or a transmembrane domain at the C-terminus and was predicted to be located in the extracellular space by WoLF PSORT and TargetP programs. Our experimental evidence from transient expression of the GFP-tagged Sl3-MMP in onion epidermal cells clearly demonstrated that Sl3-MMP is targeted to plasma membrane but not secreted into the apoplast (Fig. 11). This is consistent with the previous observations that both of soybean Slti114 and tobacco Nt1-MMP, which are phylogenetically closest MMPs to Sl3-MMP, localized in the plasma membrane [11, 34], but differs from the soybean SMEP1, which was reported to be tightly bound to the cell wall [21]. Animal MMPs have been recognized as a class of enzymes that plays a critical role in ECM turnover and remodeling based on their ability to hydrolyze the major protein components of the ECM [35]. However, whether Sl3-MMP exerts its biological function through degradation of plant ECM components needs to be further investigated.

Direct evidence supporting the involvement of MMPs in plant disease resistance is lacking although members of the MMP family have been shown to be induced by pathogen infection and defense signaling hormones [2, 20]. In the present study, we observed that the expression of *Sl-MMPs* could be induced by both *Pst* DC3000 and *B. cinerea* (Figs. 3 and 4). This is similar to the observations that the expression of *Gm2-MMP* in soybean [19] and *Nt1-MMP* in tobacco suspension cells [11] was induced by different fungal, oomycetes or bacterial pathogens. However, pathogen-induced expression patterns varied among the *Sl-MMPs*, e.g., all *Sl-MMPs*, especially for *Sl1-MMP*, *Sl3-MMP* and *Sl5-MMP*, exhibited upregulated expression patterns in response to *B. cinerea* while only *Sl1-MMP*, *Sl2-MMP* and *Sl3-MMP* showed upregulated patterns in response to *Pst* DC3000 (Fig. 2). In addition, the expression of most of the *Sl-MMPs* was also induced by defense signaling hormones such as SA, JA and ACC (Fig. 3). This is consistent with *At2-MMP* whose expression was induced rapidly by MeJA [18] but differs from *Gm2-MMP*, which was not induced by SA and MeJA in soybean suspension cells [19]. Therefore, it is likely that the expression of *Sl-MMP*s is precisely controlled by complex mechanisms in response to infection from different pathogens and/or defense signaling hormones.

Our VIGS- and transient expression-based functional analyses led to the identification of Sl3-MMP as a positive regulator of defense response against *B. cinerea*. Firstly, silencing of *Sl3-MMP* but not other four *Sl-MMPs* resulted in reduced resistance against *B. cinerea* and *Pst* DC3000, as the *Sl3-MMP*-silenced plants exhibited severe disease symptoms and supported more *in planta* pathogen growth (Figs. 6 and 7). Accompanying with the reduced resistance in *Sl3-MMP*-silenced plants was attenuated defense responses upon infection of *B. cinerea*, e.g., increased ROS accumulation and cell death and downregulated expression of JA/ET-mediated signaling responsive defense-related genes (Figs. 8 and 9). *B. cinerea* can induce the generation of ROS in plants to benefit its infection [36–38]; however, ROS may function in different ways in the interaction between tomato and *B. cinerea* [39]. It is generally accepted that ROS accumulated during the late stage directly benefits the establishment of infection by *B. cinerea* [39] and that sustained production of ROS as a facilitator of cell death may promote susceptibility [40]. In the present study, the *Sl3-MMP*-silenced plants accumulated larger amount of H_2_O_2_ at 24 h than the control plants after inoculation (Fig. 7a and b). This increase in ROS accumulation in the *Sl3-MMP*-silenced plants was mainly due to an accelerated ROS generation rather than a reduced ROS scavenging ability because the expression levels of *SlRboh1* and *SlWfi1*, coding for NADPH oxidases that are plasma membrane-localized ROS generating enzymes [41], were significantly higher but the expression levels of *SlCAT and SlSOD*, coding enzymes capable of scavenging ROS [42] were similar to those in the control plants after infection of *B. cinerea* (Fig. 8a). Increased expression levels of *SlAPX* and *SlGR* in the *Sl3-MMP*-silenced plants might be responses to the change in cellular redox status caused by the excessive ROS accumulated after infection of *B. cinerea*. Furthermore, excessive ROS accumulation in the *Sl3-MMP*-silenced plants thus resulted in an increased level of cell death, as revealed by Trypan blue staining and measurement of electrolyte leakage (Fig. 7c and d), which should favor the growth of *B. cinerea* (Fig. 5d). It is therefore likely that accelerated ROS accumulation in *B. cinerea*-infected *Sl3-MMP*-silenced plants may be one of the mechanisms leading to a reduced resistance to *B. cinerea*. On the other hand, *B. cinerea*-induced expressions of *SlPR1a* and *SlPR1b*, regulated by the SA-mediated signaling pathway [27], and *SlLapA*, *SlPI-I*, and *SlPI-II*, regulated by the JA/ET-mediated signaling pathway [27], were significantly increased and suppressed, respectively, in *Sl3-MMP*-silenced plants (Fig. 9b), indicating that silencing of *Sl3-MMP* may affect the efficiency of the JA/ET-mediated signaling pathway in regulating expression of defense-related genes. Secondly, transient expression of *Sl3-MMP* in *N. benthamiana* plants conferred an enhanced resistance to *B. cinerea* and upregulated expression of defense genes (Fig. 9), further supporting the hypothesis that Sl3-MMP acts as a positive regulator of defense response against *B. cinerea*. This is consistent with the observations that ectopic overexpression of *MtMMPL1* led to numerous abortive infections and an overall decrease in the number of nodules upon *Sinorhizobium meliloti* infection [13]. Additionally, silencing of *Sl3-MMP* also led to a reduced resistance to *Pst* DC3000, a hemibiotrophic bacterial pathogen that has different life style from that of *B. cinerea*, indicating a broad involvement of Sl3-MMP in regulation of disease resistance against different pathogens.

## Conclusion

Tomato genome encodes five *Sl-MMP* genes and all of them exhibited differentially expression patterns in response to pathogens and defense signaling hormones. The present study focused on the characterization of Sl-MMPs in disease resistance and data from VIGS- and transient expression-based analyses demonstrated that Sl3-MMP functions as a positive regulator of defense response against *B. cinerea* and *Pst* DC3000 in tomato. Further biochemical studies indicate that Sl3-MMP possess an *in vitro* enzymatic activity with different cleavage site specificities and is targeted on the plasma membrane. However, the biological functions of other four Sl-MMPs need to be investigated. Further identification of endogenous substrates for Sl3-MMPs will be helpful in elucidation of the biochemical mechanism of Sl3-MMP in disease resistance.

## Methods

### Plant growth, treatment and disease assays

Tomato (*Solanum lycopersicum* L.) cv. Suhong 2003 was used for all experiments. Plants were grown in a mixture of perlite: vermiculite: plant ash (1:6:2) in a growth room under fluorescent light (200 μE m^2^ s^−1^) at 22-24 °C with 60 % relative humidity and a 14 h light/10 h dark cycle. Pathogen inoculation, disease assays and measurement of pathogen in planta growth were performed basically according to previously described protocols [43, 44]. For analysis of gene expression in responding to pathogen infection, mock-inoculation controls were set by treating the plants with buffer solution (*B. cinerea*) or 10 mM MgCl_2_ (*Pst* DC3000). For analysis of gene expression in response to defense signaling hormones, 4-week-old tomato plants were treated by foliar spraying with 100 μM MeJA, 100 μM ACC or 100 μM SA in 0.1 % ethanol and equal volume of 0.1 % ethanol solution as control. Leaf samples were collected at indicated time points after treatment or inoculation and stored at -80 °C until use.

### Identification of tomato Sl-MMPs and bioinformatics analyses

Arabidopsis AtMMPs were used as queries to perform multiple database searches against the proteome and genome files downloaded from the SOL Genomics Network (SGN, http://solgenomics.net) [45]. BlastP and TBlastN at NCBI (http://blast.ncbi.nlm.nih.gov) were performed with an e-value cutoff set to 1e − 003 [46]. All protein sequences were compared with known MMP sequences using ClustalX (http://www.clustal.org/) to verify the sequences were candidate MMPs. The obtained MMP sequences were examined by domain analysis programs PFAM (http://pfam.sanger.ac.uk/) and SMART (http://smart.embl-heidelberg.de/) with the default cutoff parameters [47, 48]. The isoelectric points and molecular weights were predicted with the help of the proteomics and sequence analysis tools on the ExPASy Proteomics Server (http://expasy.org/). Sequence alignment was carried out by the ClustalX program [49]. Putative signal peptides and transmembrane domains were predicted by SignalP 4.1 (http://www.cbs.dtu.dk/services/SignalP/) and TMpred (http://ch.embnet.org/software/TMPRED_form.html), respectively. Phylogenetic trees including the tomato MMP protein sequences were constructed using the neighbor-joining (NJ) method of the MEGA6 program with the p-distance and complete deletion option parameters [50]. The reliability of the obtained trees was tested using a bootstrapping method with 1000 replicates.

### Cloning of the *Sl-MMP* genes

The coding sequence of the *Sl3-MMP* gene was amplified using a pair of gene-specific primers (Additional file 3) from tomato cDNAs and cloned into pMD19-T vector, yielding plasmids pMD19-Sl-MMPs. After confirmation by sequencing, the plasmid pMD19-Sl3-MMP was used for further experiments.

### Purification of recombinant Sl3-MMP protein and proteolytic activity assays

The coding sequence for the mature form of Sl3-MMP protein (Sl3-MMPm) was amplified from pMD19-Sl3-MMP with a pair of primers (Additional file 3) and cloned into pGEX-4 T-3 vector at *Eco*RI/*Xho*I sites, which was fused to glutathione-S-tranferase (GST) at its N-terminal. The recombinant plasmid pGEX-Sl3-MMPm and empty vector were introduced into the *E. coli* strain Rosetta DE3 and expression of Sl3-MMPm fusion and GST tag in *E. coli* cells was induced by 1 mM isopropyl-D-thiogalactoside (IPTG) at 20 °C overnight. The GST-tagged Sl3-MMPm fusion protein and GST tag were purified using the Bug-Buster GST-Bind purification kit following the manufacturer’s protocols (Merck, Darmstadt, Germany). Protein concentration was determined using Bio-Rad protein assay kit (Bio-Rad, CA, USA) following the recommended method.

For proteolytic activity assays, purified Sl3-MMPm or GST (a negative control) was incubated with 5 μg of MBP (Sigma-Aldrich, St. Louis, MO, USA) in the absence (-) or presence (+) of 1 mM EDTA in 200 mM Tris–HCl, pH7.5, containing 10 mM CaCl_2_, 0.1 % (w/v) Brij35 and 1× EDTA-free protease inhibitor cocktail (Roche Diagnostics, Mannheim, Germany) at 37 °C overnight. Products were analyzed by 16 % Tricine–SDS-PAGE and stained in Colloidal Coomassie [51].

Proteolytic activity of Sl3-MMP were measured using the synthetic fluorescent substrates Mca-Pro-Leu-Gly-Leu-Dpa-Ala-Arg-NH2 (QF-24), Mca-Pro-Leu-Ala-Nva-Dpa-Ala-Arg-NH2 (QF-35) and Mca-KESAbuNLFVLKDpaR-NH2 (QF-75) (synthesized by Invitrogen Life Technologies, Inc.). Purified recombinant Sl3-MMPm was incubated to a final concentration of 1 μM in a total volume of 100 μL of 50 mM Hepes, pH7.5, 5 mM CaCl_2_, and 10 μM ZnCl_2_. Synthetic quenched fluorescent peptide substrates were added from a 100x stock in DMSO to a final concentration of 1 μM. The excitation and emission wavelengths were set at 320 and 405 nm, respectively and the fluorescence was measured at one minute intervals for 90 min.

### Subcellular localization

The coding sequence of *Sl3-MMP* was amplified from pMD19-Sl3-MMP using a pair of primers Sl3-MMP-GFP-F and Sl3-MMP-GFP-R (Additional file 3) and inserted into pFGC-Egfp at *Bam*HI/*Xba*I sites. The recombinant plasmid pFGC-Sl3-MMP and the empty vector pFGC-Egfp were introduced into onion epidermal cells by particle bombardment method. Particle bombardment was performed with a PDS-1000 (Bio-Rad, Hercules, CA, USA) according to the manufacturer’s instructions. GFP was detected 24 h after bombardment. Plasmolysis was achieved by treating the bombarded onion epidermal cells with 0.8 M mannitol for 10 min. Microscopic observation was performed using a Zeiss LSM 780 confocal laser scanning microscope (Carl Zeiss, Germany) and representative photographs were taken.

### VIGS in tomato and transient expression in *N. benthamiana*

For VIGS constructs, fragments of 300-400 bp (Additional file 2) for *Sl-MMPs* were amplified from tomato cDNAs using gene-specific primers (Additional file 3) and cloned into pTRV2 vector [26], yielding pTRV2-Sl1-MMP-pTRV2-Sl5-MMP*.* The recombinant plasmids with pTRV2-GUS (as control) were then introduced into *Agrobacterium tumefaciens* strain GV3101 by electroporation using GENE PULSER II Electroporation System (Bio-Rad Laboratories, Hercules, CA, USA). Agrobacteria carrying pTRV2-GUS (control) or pTRV2-Sl-MMP plasmids were grown in YEP medium (50 μg/ml rifampicin, 50 μg/ml kanamycin and 25 μg/ml gentamicin) for 24 h with continuous shaking at 28 °C. Cells were centrifuged and resuspended in infiltration buffer (10 mM MgCl_2_, 10 mM MES, 200 μM acetosyringone, pH5.7). Agrobacteria carrying pTRV2-GUS or pTRV2-Sl-MMP were mixed with agrobacteria carrying pTRV1 in a ratio of 1:1 and adjusted to OD_600_ = 1.5. The mixed agrobacteria suspension was infiltrated into the abaxial surface of 2-week-old seedlings using a 1 ml needleless syringe. Efficiency of the silencing protocol was examined using phytoene desaturase (PDS) gene as a marker of silencing in tomato plants according to the protocol described previously [26]. The VIGS-infiltrated plants were allowed to grow for three weeks under same condition as mentioned above and then used for all experiments.

For transient expression in *N. benthamiana*, agrobacteria carrying pFGC-Sl3-MMP or pFGC-eGFP empty vector were infiltrated into leaves of 4-week-old plants using 1 ml needleless syringes. Leaf samples were collected 2 days after agroinfiltration for analyzing the expression level of *Sl3-MMP* and were used for disease assays and physiological, biochemical and molecular analyses.

### qRT-PCR analysis of gene expression

Total RNA was extracted by Trizol regent (TaKaRa, Dalian, China) according to the manufacturer’s instructions. RNA was treated with RNase-free DNase and then reverse-transcribed into cDNA using the PrimeScript RT regent kit (TaKaRa, Dalian, China). The obtained cDNAs were used for gene expression analysis with real time quantitative PCR. Each qPCR reaction contained 12.5 μL SYBR Premix Ex Taq (TaKaRa, Dalian, China), 0.1 μg cDNA and 7.5 pmol of each gene-specific primer (Additional file 3) in a final volume of 25 μL, and had three independent biological replicates. The qPCR was performed in a CFX96 real-time PCR detection system (BioRad, Hercules, CA, USA). Relative gene expression level was calculated using 2^–△△CT^ method as described [52].

### Western blot analysis

Leaf discs were ground into 200 μl lysis buffer (50 mM Tris–HCl, pH7.4, 150 mM NaCl, 1 mM EDTA, 1 mM DDT, 0.1 % (*v/v*) Triton X-100, and 1× protease inhibitor cocktail from Sigma plus 1 mM PMSF), followed by addition of 100 μl loading buffer. The samples were boiled for 5 min and subsequently centrifuged at 10,000× *g* for 10 min at 4 °C. Proteins in 20 μL of the supernatant were separated on a 12 % SDS-PAGE gel and transferred onto PVDF membrane by semi-dry transfer. Detection of GFP was performed using a polyclonal rabbit anti-GFP antibody (1:5000 dilution; GenScript, Nanjing, China) and a Horseradish peroxidase-conjugated anti-rabbit antibody (1:10,000 dilution; GenScript, Nanjing, China) according to the manufacturer’s instructions. Proteins on PVDF membranes were detected by SuperSignal West Pico Chemiluminescent Substrate (Thermo Scientific, Rockford, IL, USA).

### Histochemical assays and measurement of electrolyte leakage

Detection of H_2_O_2_ was performed by DAB staining [53]. Leaf samples were collected from inoculated tomato plants at 24 h after inoculation or *N. benthamiana* plants at 48 h after infiltration for transient expression. Leaves were dipped into DAB solution (1 mg/ml, pH3.8) and incubated for 8 h in dark at room temperature. The DAB-treated leaves were removed, placed into acetic acid/glycerol/ethanol (1:1:1, vol/vol/vol), and boiled for 5 min in a water bath, followed by several changes of the solution. Subsequently, the leaves were maintained in 60 % glycerol. Accumulation of H_2_O_2_ was visualized using a digital camera and quantified using ImageJ software (National Institutes of Health) from DAB image. Trypan blue staining to visualize cell death and *B. cinerea* hypha were performed as previously described [44]. Fresh tissue was harvested, stained, and boiled for 30 s in lactophenol (10 mL of lactic acid, 10 mL of glycerol, 10 mL of liquid phenol, and 10 mL of distilled water) containing 10 mg of trypan blue. Tissue was rapidly transferred and boiled in alcoholic lactophenol (2:1 95 % ethanol:lactophenol) for 1 min, washed in 50 % ethanol at room temperature for 2 min, and stored in water. The stained leaves were examined using a digital camera.

Electrolyte leakage was measured as previously described [54]. Leaf discs (0.5 cm diameter) were washed in sterile double-distilled water for 30 min, followed by incubation for 2 h at room temperature with gentle agitation. Electrolyte leakage from the leaf samples was evaluated by measuring ion conductivity using a conductivity meter (FE30, Mettler-Toledo Group, Switzerland).

### Statistical analysis

All experiments were repeated independently three times. Data obtained from three independent experiments were subjected to statistical analysis according to the Student’s *t*-test and the probability values of *p* < 0.05 were considered as significant difference.

### Accession numbers for *Sl-MMPs* and the defense-related genes

The *Sl-MMP* sequences were deposited in GenBank database under the following accession numbers: Sl1-MMP, KR081423; Sl2-MMP, KR081424; Sl3-MMP, KR081425; Sl4-MMP, KR081426; Sl5-MMP, KR081427. The defense-related genes used in this study and their GenBank accession numbers are as follows: *SlRboh1*, respiratory burst oxidase homolog 1 (NM_001247197); *SlWfi1*, whitefly-induced 1 (NM_001247342); *SlAPX*, ascorbate peroxidase (XM_006366063); *SlGR*, glutathione reductase (XM_010328522); *SlCAT*, catalase (XM_004238382); *SlSOD*, superoxide dismutase (AF527880); *PR1a*, pathogenesis-related 1a (NM_001247869); *PR1b*, pathogenesis-related 1b (NM_001247385); *SlLapA*, leucine aminopeptidase (AK319505); *SlPI-I*, proteinase inhibitors I (M13938); *SlPI-II*, proteinase inhibitors II (JN091682).

### Availability of supporting data

The cDNA and protein sequences of Sl-MMPs and the VIGS fragments for Sl-MMP genes used in this study are included in Additional files 1 and 2. Phylogenetic datasets are available for download at Dryad (http://datadryad.org/; doi: 10.5061/dryad.7qh1r).

## Additional files

Additional file 1:
**Sequences of cDNAs and proteins of Sl-MMPs.**
Additional file 2:
**Sequences of the VIGS fragments for** ***Sl-MMPs***. Additional file 3: Table S1.
**Primers used in this study for different purposes.**

## Abbreviations

ACC: 1-amino cyclopropane-1-carboxylic acid
APX: Ascorbate peroxidases
*B. cinerea*: *Botrytis cinerea*
CAT: Catalases
DAB: 3, 3-diaminobenzidine
dpi: Days post-inoculation
ECM: Extracellular matrix
EDTA: Ethylenediaminetetraacetic acid
GR: Glutathione reductase
MeJA: Methyl jasmonate
MMP: Matrix metalloproteinase
PDS: Phytoene desaturase
*Pst*: *Pseudomonas syringae* pv. *tomato*
QF: Quenched fluorescent
ROS: Reactive oxygen species
qRT-PCR: Quantitative reverse transcription PCR
SA: Salicylic acid
SOD: Superoxide dismutase
VIGS: Virus-induced gene silencing
