# Supplementary material for: Tomato Sl3-MMP, a member of the Matrix metalloproteinase family, is required for disease resistance against Botrytis cinerea and Pseudomonas syringae pv. tomato DC3000
Source: BMC Plant Biol. 2015 Jun 14;15:143. doi: 10.1186/s12870-015-0536-z (PMC4465618; doi:10.1186/s12870-015-0536-z)
Supplement: Additional file 1: — Sequences of cDNAs and proteins of Sl-MMPs. [file 12870_2015_536_MOESM1_ESM.doc]

Additional file 1

**Sequences of cDNAs and proteins of Sl-MMPs.**

**Sl1-MMP (Solyc08g078550)**

**CDS** ATGTCCCCGTTTCCAAATTATTTGTATTTCACTTTTGCTCTGTTCCTTCTCCTCCTTTCGCTCCCATCTTTTCCCGCCAGAATATTACAACAGGATCCGTTGACGGAACTCGCCGCCGATATTCAAAACAACACGTGGCATGCTTTCGTTAAATTAATCGACGCCGGAAAAGGCAGTCAGGTCACCGGCATGTCGGAGCTGAAAAAATACTTCCAGCGATTCGGCTATATGCCAATTCCCGATCAAAATTTCACCGATTTTTTCGACGAGGACTTAGAGTCTGCAGTACTCAATTATCAGAAAAACCTCGGTTTACCAGTCACCGGAAAACTAGACGATGAAACGATGAGTGAAATTATGCTTCCGCGTTGCGGTATGAGCGATTTAGTTCATGATCACAGCTCGTTACATACTACGAAAAATTACGCTTACTTTTACGGAAGACCTAGGTGGATGAAAACATCTCCGATGATTTTATCTTACGCTTTTTCACCGAATTCGATGATCGATTACATCGACGCATCGGAAATTAGATCCGTTTTTCAACGAGCGTTTGCGCGGTGGTCTTCTGCGATTCCAGTGAATTTCACTGAAACAGAGGATTACTTCACAGCGGATATTAAAATCGGTTTCTACAGCGGCGATCACGGCGACGGAGAACCGTTTGACGGAGTGCTGGGAGTGTTAGCTCACGCTTTTTCGCCGGAGAACGGGAGGTTCCATTTAGACGCGGCGGAAACATGGGCCGTTGATTTCGATGAAGAAGGATCAAAAGTGGCTGTGGATTTAGAATCAGTAGCGACCCATGAGATTGGGCATGTACTTGGGCTTGCTCATTCATCGGTAAAAGATGCAGTAATGTACCCAAGTTTAAGCCCAAGAACGAAGAAAAGGGATTTGAAGCTTGATGACGTGGAAGGGGTCCAAGCTTTATATGGGTCAAACCCGAATTTTAAGTACACGTCATCATTGGAACATGACACGTCATCATCAAATAGAAGAAGAAGAACAACATCAAAGTGGACCACTTTTTTGGGAATGGTGGTTTTGATATTTTCCTTGTGTTTATGA

**Protein**

MSPFPNYLYFTFALFLLLLSLPSFPARILQQDPLTELAADIQNNTWHAFVKLIDAGKGSQVTGMSELKKYFQRFGYMPIPDQNFTDFFDEDLESAVLNYQKNLGLPVTGKLDDETMSEIMLPRCGMSDLVHDHSSLHTTKNYAYFYGRPRWMKTSPMILSYAFSPNSMIDYIDASEIRSVFQRAFARWSSAIPVNFTETEDYFTADIKIGFYSGDHGDGEPFDGVLGVLAHAFSPENGRFHLDAAETWAVDFDEEGSKVAVDLESVATHEIGHVLGLAHSSVKDAVMYPSLSPRTKKRDLKLDDVEGVQALYGSNPNFKYTSSLEHDTSSSNRRRRTTSKWTTFLGMVVLIFSLCL

**Sl2-MMP (Solyc04g005040)**

**CDS** ATGAGGAGGATTCATCTATACATCGCCATTGCTTATGTTGTAATATTTCAAACTAGTTGTTCAGCTCATTTCTTTCCAAATATTTCATCAATCCCTTCTTCATTACTCAAACCTAATGCCACTGCTTGGACTTCTTTTCAAAAGTTGTTAGGATGTCAACCTGGTCAGAAAGTCGATGGCATTGCTAAAATCAAAAAATATTTTCAACATTTTGGTTACATTAATAATTTGACTAGTTTTAATTTCACTGATGAATTTGATGATACTCTTGAATCTGCTCTCAAGACGTATCAGAGAAATTTCAACCTCAAAGCCACCGGTGTGCTCGATGCGCCCACCATTCAGCATCTCATAAAACCCAGATGTGGAAATGCCGATCTAGTTAACGGTACTAGTACTATGAACGCTGGAAAGCCGCACACGGTGGCTCACTACTCCTTCTTTCCTGGAAGACCAAAGTGGCCCGAGGGTAAGACTGATTTGACTTATGCCTTTCTACCGGCAAACAATTTGACGGATGATATTAAGAGTGTCTTCTCACGTGCGTTTGATCGGTGGTCGGAGGTAACCCCGTTGAGCTTCACGGAAATACCGTCATTTCAATCGGCTGATATCAAGATCGGATTTTTAACCGGAGATCACAATGATGGAGAGCCGTTTGATGGTCCGATGGGGACATTAGCGCACGCGTTTTCACCGCCGGCGGGGCATTTTCACTTGGACGGCGAGGAAAATTGGGTCGTCGACGGTGTGCCGGTAAATGAAGGGAACTTTTTTTCTATATTGTCGGCTGTGGATCTCGAATCGGTTGCGGTTCATGAAATCGGGCATTTATTGGGTTTGGGTCATTCATCGGTAGAAGATTCGATTATGTATCCGAGTTTAGAATCGGGTATTCGAAGAGTGGAGCTGGTGGAGGATGATATTAAGGGGGTTCAGGAATTATACGGGTCAAACCCGAATTATACTGGGACCAATACAACATTGACTCCGAGCGGCCTGGATAATGATACGAATGGAGCCCCGATTCGTAGCTCAGTATGGATTCATGGGTTTTTATTGGTGGTTGGATTTTTCATTTATTCAATTTAA

**Protein**

MRRIHLYIAIAYVVIFQTSCSAHFFPNISSIPSSLLKPNATAWTSFQKLLGCQPGQKVDGIAKIKKYFQHFGYINNLTSFNFTDEFDDTLESALKTYQRNFNLKATGVLDAPTIQHLIKPRCGNADLVNGTSTMNAGKPHTVAHYSFFPGRPKWPEGKTDLTYAFLPANNLTDDIKSVFSRAFDRWSEVTPLSFTEIPSFQSADIKIGFLTGDHNDGEPFDGPMGTLAHAFSPPAGHFHLDGEENWVVDGVPVNEGNFFSILSAVDLESVAVHEIGHLLGLGHSSVEDSIMYPSLESGIRRVELVEDDIKGVQELYGSNPNYTGTNTTLTPSGLDNDTNGAPIRSSVWIHGFLLVVGFFIYSI

**Sl3-MMP (Solyc04g005050)**

**CDS** ATGAGGATTCCTTTATTCATCGCCATACTTTTTGTTCTTAGTGTTCCATTTCCATCTTCAGCTCATTTCTTCCCAAATATTTCTTCAATTCCTCCTAATTTATTGAAACCAAATGCCACTGCCTGGGATGCTTTTAACAAGTTATTAGGATGCCATTCCGGTCAGACGGTCGACGGCTTAGCGAAAATCAAAAAATATTTTCACTACTTTGGATACATTAATAATTCTTCCACTAACTTCACTGATGATTTTGATGATACTCTTGAATCTGCTCTCAAGACCTACCAGCTTAACTTCAACCTCAACACCACCGGTGTGCTCGACGCGAACACCATTCAGCATCTCATAAAACCCAGATGTGGAAACGCTGATGTAGTTAACGGTACTAGTACTATGAACTCCGGTAAGCCACCGGCAGGTTCTCCGACGATGCACACCGTAGCTCACTACTCCTTCTTTCCGGGAAGTCCACGGTGGCCGGCGAACAAGAGAGATCTGACATATGCTTTTGCACCGCAGAATGGACTGACGGATGATATTAAGATTGTGTTCACACGAGCGTTTGATAGGTGGTCGGAGGTGACTCCATTGACGTTTACTGAAATAGCATCGTACCAATCGGCTGATATTAAGATCGGGTTTTTCAGCGGAGATCACAACGATGGAGAGCCGTTTGATGGTCCTATGGGGACATTAGCACACGCGTTTTCCCCACCGGCGGGGCATTTTCACTTGGACGGCGAGGAGAACTGGGTGATCGACGGTGCGCCGATAGTTGATGGGAATTTCTTTTCTATATTGTCGGCGGTGGACCTTGAATCGGTTGCGGTTCATGAAATCGGGCATTTATTGGGTTTGGGTCATTCATCCGTAGAAGATGCTATTATGTACCCGACTTTAGGAGCGGGTACCCGAAGAGTCGAGCTTAGAAATGATGATATATTGGGAGTCCAGGAGTTATACGGGTCTAACCCGAATTATACTGGGCCAAACCCAAATTTGACTCCGAGCCAAGAGAGTGACACAAATGGAGCCCCGATATTTGAGTTATCATGGTTTCATGGGTTTCTTGGTTTATTCTTTGCTTTGTTCATTCAACTGTAG

**Protein**

MRIPLFIAILFVLSVPFPSSAHFFPNISSIPPNLLKPNATAWDAFNKLLGCHSGQTVDGLAKIKKYFHYFGYINNSSTNFTDDFDDTLESALKTYQLNFNLNTTGVLDANTIQHLIKPRCGNADVVNGTSTMNSGKPPAGSPTMHTVAHYSFFPGSPRWPANKRDLTYAFAPQNGLTDDIKIVFTRAFDRWSEVTPLTFTEIASYQSADIKIGFFSGDHNDGEPFDGPMGTLAHAFSPPAGHFHLDGEENWVIDGAPIVDGNFFSILSAVDLESVAVHEIGHLLGLGHSSVEDAIMYPTLGAGTRRVELRNDDILGVQELYGSNPNYTGPNPNLTPSQESDTNGAPIFELSWFHGFLGLFFALFIQL

**Sl4-MMP (Solyc05g006360)**

**CDS** ATGAGAATTTTTCTATTCTCCCTCGTTATAATTGTTGCTTTAATAATCGTCGATTCATCTTCCCCTGTTTCAGCTCATGTTTCCCCTGTTTCTGCTCATTTTTACGATAACGTTCGTAATTCTACCTGGAACTATTTCAACAACTACTTGGGTTGCCGCGTTGGGCAAAAGATTAAAGGATTAGCGAAAATCAAACAGTATTTTCAACGTTTTGGGTATATTGATGATTCTTTGAGCAATGATTTTACAGATGAATTTGATCAACTTCTTTTATCAGCTCTGAAGAGTTATCAATTAAACTTTAATCTTAACGTAACCGGAGAATTCGACTTCTCCACTCTTCAAAATATGGTAAAACCAAGATGCGGTAATCCAGATATAGCAAAAAAGGGCTACGGGGGCAAAACTCCAATGGATCATACAGTGGCACATTTCTCGTTTTTCGAAGGCCAGCGACGTTGGCCTTCGAGTAAGAGTAAATTAAAGTATGCATTTCTACCGGAGAATCAGTTGACGGATTCTGTTAAGTTAGCTTTTAGGAGGGCGTTTGATAAATGGTCAAAAGTAACACCGTTAACTTTTAAAGAGATGGGTTCTTATAGATCCGCGGATATTCGGATAGGGTTTTTTGTCCGAGACCATGGAGATGGTAACCCGTTCGATGGACCCATGAAGGTTTTGGCACACGCGTTTGCACCTCCAATAGGGTTTTTCCACTTAGATGGCGAGGAAAATTGGATGGTTGACGGCGAGTATTTGAAAGAAGGGATGGTGGATCTTGAATCAGTTGCGGTTCATGAAATCGGGCATTTATTGGGTTTGGATCATTCGTTCGAAAAAGACGCGGTTATGTTTCCGACACTTGAAGATGGAACGAGGAAAGTGGAGTTGTCAAGAGATGATATTGAAGGGGTACAAATGTTATATGGGTCAAACCCGGATTATAATGGGTCAAGTACGGTTTACACACACCATCAAGAGAATGATATTAGTGGATACTCCACTTTTCGTTCATTGTGCCCACATTGGCTTGTTGGATTTTTCTTAGCATTGGTGCTTTCGATTTCTCTATAG

**Protein**

MRIFLFSLVIIVALIIVDSSSPVSAHVSPVSAHFYDNVRNSTWNYFNNYLGCRVGQKIKGLAKIKQYFQRFGYIDDSLSNDFTDEFDQLLLSALKSYQLNFNLNVTGEFDFSTLQNMVKPRCGNPDIAKKGYGGKTPMDHTVAHFSFFEGQRRWPSSKSKLKYAFLPENQLTDSVKLAFRRAFDKWSKVTPLTFKEMGSYRSADIRIGFFVRDHGDGNPFDGPMKVLAHAFAPPIGFFHLDGEENWMVDGEYLKEGMVDLESVAVHEIGHLLGLDHSFEKDAVMFPTLEDGTRKVELSRDDIEGVQMLYGSNPDYNGSSTVYTHHQENDISGYSTFRSLCPHWLVGFFLALVLSISL

**Sl5-MMP (Solyc10g018750)**

**CDS** ATGTCTATCTTCTTTAATTTCTTCCTTGCTTCTTTTCTCTTCTTCTCTCCATGCCTTTCTATTAGAACAACAACATTTTTTCACTCCAAAACTATTAATCACACAAACCATCATCATAAAATTCAAACTTACACATGGCATAAGTTTAAAATTTTTGTGGATGCTACAAAAGGCAAGAAAATCAATGGAATTTCTGAGCTCAAGAAATATTTTCATCGATTCGGATATATGAAGATGGATTATAATAACTTGAACTTCACAGATTTATTTGATGATCACTTAGAACATGCCTTGATTAAATATCAAGAAAAGTTGGGCCTTTTAGTCACTGGAAAACTTGATGAAAATACAGTTTCTCAGATTATTTCACCTAGATGTGGCGTATCCGATTCTACGCCTCAATTATTCATGCATGCAAAGAGAAACTATGCATTTTTCACAGGCAGACCAAGATGGTCTAGAAGTATACCAATAACATTAACTTATGCATTCTCTAAAGAGTATGTAATAAGTTCATTGAGCATGTTGGAGATAAAGGATGCTTTTCAACGCGCGTTCAACCATTGGGCATTGGTAATACCGGTTACATTCATGGGAAGCAACGATTACGGATTTGCAGACATTAGAATAGGGTTTTACAAAGGTGATCATGGAGATGGAGAGGCATTTGATGGTGTTCTAGGGGTTTTAGCTCATGCATTTTCACCAGAAACTGGTAGGTTCCATCTAGACGCGGCGGAGACATGGGCAGTGGACTTTGAAAGAGAAAAATCAGATGTAGCTATTGATTTAGAATCAGTAGCTACACATGAGATTGGACATTTGTTAGGGTTAGCACATACTTCAGTTCAAGAAGCAGTAATGTTCCCTAGTTTAAAGCCAAGAGAAAAGAAAGTTGATTTGAAAATGGATGATATTAAGGGAATACAAGCTCTTTATGGTTCAAACCCTAATTTTAGTTATAAAGCTTTATTAGAATCTGATACTTCTACTAACAATGGAGCTACTTTGATGAAGAGACAATTAACATTGTCTAATTTTATTTTAGTCTTAATCTTGTTCATGTCTATGTAA

**Protein**

MSIFFNFFLASFLFFSPCLSIRTTTFFHSKTINHTNHHHKIQTYTWHKFKIFVDATKGKKINGISELKKYFHRFGYMKMDYNNLNFTDLFDDHLEHALIKYQEKLGLLVTGKLDENTVSQIISPRCGVSDSTPQLFMHAKRNYAFFTGRPRWSRSIPITLTYAFSKEYVISSLSMLEIKDAFQRAFNHWALVIPVTFMGSNDYGFADIRIGFYKGDHGDGEAFDGVLGVLAHAFSPETGRFHLDAAETWAVDFEREKSDVAIDLESVATHEIGHLLGLAHTSVQEAVMFPSLKPREKKVDLKMDDIKGIQALYGSNPNFSYKALLESDTSTNNGATLMKRQLTLSNFILVLILFMSM
