# Supplementary material for: Tomato Sl3-MMP, a member of the Matrix metalloproteinase family, is required for disease resistance against Botrytis cinerea and Pseudomonas syringae pv. tomato DC3000
Source: BMC Plant Biol. 2015 Jun 14;15:143. doi: 10.1186/s12870-015-0536-z (PMC4465618; doi:10.1186/s12870-015-0536-z)
Supplement: Additional file 2: — Sequences of the VIGS fragments for Sl-MMPs. [file 12870_2015_536_MOESM2_ESM.docx]

Additional file 2

**Sequences of the VIGS fragments for *Sl-MMPs***

**Sl1-MMP-vigs:** 368 bp

CCGTTTGACGGAGTGCTGGGAGTGTTAGCTCACGCTTTTTCGCCGGAGAACGGGAGGTTCCATTTAGACGCGGCGGAAACATGGGCCGTTGATTTCGATGAAGAAGGATCAAAAGTGGCTGTGGATTTAGAATCAGTAGCGACCCATGAGATTGGGCATGTACTTGGGCTTGCTCATTCATCGGTAAAAGATGCAGTAATGTACCCAAGTTTAAGCCCAAGAACGAAGAAAAGGGATTTGAAGCTTGATGACGTGGAAGGGGTCCAAGCTTTATATGGGTCAAACCCGAATTTTAAGTACACGTCATCATTGGAACATGACACGTCATCATCAAATAGAAGAAGAAGAACAACATCAAAGTGGACCAC

**Sl2-MMP-vigs:** 341 bp

TATTGGGTTTGGGTCATTCATCGGTAGAAGATTCGATTATGTATCCGAGTTTAGAATCGGGTATTCGAAGAGTGGAGCTGGTGGAGGATGATATTAAGGGGGTTCAGGAATTATACGGGTCAAACCCGAATTATACTGGGACCAATACAACATTGACTCCGAGCGGCCTGGATAATGATACGAATGGAGCCCCGATTCGTAGCTCAGTATGGATTCATGGGTTTTTATTGGTGGTTGGATTTTTCATTTATTCAATTTAATAAATAACAGCTGTACTTTTTGTTCCCTTTATATGAGATTGTATGGTATCATTGTGGGTATGGATAGATATTTATTTCGGG

**Sl3-MMP-vigs:** 322 bp

TTGTCGGCGGTGGACCTTGAATCGGTTGCGGTTCATGAAATCGGGCATTTATTGGGTTTGGGTCATTCATCCGTAGAAGATGCTATTATGTACCCGACTTTAGGAGCGGGTACCCGAAGAGTCGAGCTTAGAAATGATGATATATTGGGAGTCCAGGAGTTATACGGGTCTAACCCGAATTATACTGGGCCAAACCCAAATTTGACTCCGAGCCAAGAGAGTGACACAAATGGAGCCCCGATATTTGAGTTATCATGGTTTCATGGGTTTCTTGGTTTATTCTTTGCTTTGTTCATTCAACTGTAGTTGATGGCATGATGTG

**Sl4-MMP-vigs:** 386 bp

TCGCAAACTTGAAATGAGAATTTTTCTATTCTCCCTCGTTATAATTGTTGCTTTAATAATCGTCGATTCATCTTCCCCTGTTTCAGCTCATGTTTCCCCTGTTTCTGCTCATTTTTACGATAACGTTCGTAATTCTACCTGGAACTATTTCAACAACTACTTGGGTTGCCGCGTTGGGCAAAAGATTAAAGGATTAGCGAAAATCAAACAGTATTTTCAACGTTTTGGGTATATTGATGATTCTTTGAGCAATGATTTTACAGATGAATTTGATCAACTTCTTTTATCAGCTCTGAAGAGTTATCAATTAAACTTTAATCTTAACGTAACCGGAGAATTCGACTTCTCCACTCTTCAAAATATGGTAAAACCAAGATGCGGTAATC

**Sl5-MMP-vigs:** 383 bp

TTCATAAATTATATGTAAAGTAAAAAATTAATTGAATCAGCAATTCAACAAAATTAGTTCCTACTCATTTCGCAAACTTGAAATGAGAATTTTTCTATTCTCCCTCGTTATAATTGTTGCTTTAATAATCGTCGATTCATCTTCCCCTGTTTCAGCTCATGTTTCCCCTGTTTCTGCTCATTTTTACGATAACGTTCGTAATTCTACCTGGAACTATTTCAACAACTACTTGGGTTGCCGCGTTGGGCAAAAGATTAAAGGATTAGCGAAAATCAAACAGTATTTTCAACGTTTTGGGTATATTGATGATTCTTTGAGCAATGATTTTACAGATGAATTTGATCAACTTCTTTTATCAGCTCTGAAGAGTTATCAATTAAACT
