# Supplementary material for: Tomato Sl3-MMP, a member of the Matrix metalloproteinase family, is required for disease resistance against Botrytis cinerea and Pseudomonas syringae pv. tomato DC3000
Source: BMC Plant Biol. 2015 Jun 14;15:143. doi: 10.1186/s12870-015-0536-z (PMC4465618; doi:10.1186/s12870-015-0536-z)
Supplement: Additional file 3: Table S1. — Primers used in this study for different purposes. [file 12870_2015_536_MOESM3_ESM.docx]

Additional file 3

**Table S1 Primers used in this study for different purposes**

| Primers | Sequences (5’-3’) | Size (bp) |
| --- | --- | --- |
| ***Cloning of cDNA*** | | |
| Sl1-MMP-F | ATGTCCCCGTTTCCAAATTATT | 1071 |
| Sl1-MMP-R | TCATAAACACAAGGAAAATATC |  |
| Sl2-MMP-F | ATGAGGAGGATTCATCTATACA | 1092 |
| Sl2-MMP-R | TTAAATTGAATAAATGAAAAAT |  |
| Sl3-MMP-F | ATGAGGATTCCTTTATTCATCG | 1104 |
| Sl3-MMP-R | CTACAGTTGAATGAACAAAGCA |  |
| Sl4-MMP-F | ATGAGAATTTTTCTATTCTCCC | 1074 |
| Sl4-MMP-R | CTATAGAGAAATCGAAAGCACC |  |
| Sl5-MMP-F | ATGGGAAGCAACGATTACGGAT | 1074 |
| Sl5-MMP-R | TTACATAGACATGAACAAGATT |  |
| ***VIGS constructs*** | | |
| Sl1-MMP-VIGS-F | TGC TCTAGA CCGTTTGACGGAGTGCTG | 368 |
| Sl1-MMP-VIGS-R | CCG GAGCTC GTGGTCCACTTTGATGTTGTTC |  |
| Sl2-MMP-VIGS-F | TGC TCTAGA TATTGGGTTTGGGTCATT | 341 |
| Sl2-MMP-VIGS-R | CCG GAGCTC CCCGAAATAAATATCTATCC |  |
| Sl3-MMP-VIGS-F | TGC TCTAGA TTGTCGGCGGTGGACCTT | 322 |
| Sl3-MMP-VIGS-R | CCGGAGCTC CACATCATGCCATCAACTACAG |  |
| Sl4-MMP-VIGS-F | TGC TCTAGA TCGCAAACTTGAAATGAG | 386 |
| Sl4-MMP-VIGS-R | CCG GAGCTC GATTACCGCATCTTGGTT |  |
| Sl5-MMP-VIGS-F | TGC TCTAGA ATGTCTATCTTCTTTAATTTCTTC | 383 |
| Sl5-MMP-VIGS-R | CCG GAGCTC ACGCCACATCTAGGTGAAATAA |  |
| ***Transient expression and subcellular localization*** | | |
| Sl3-MMP-GFP-F | GCTCTAGA ATGAGGATTCCTTTATTCATCG | 1104 |
| Sl3-MMP-GFP-R | TCCCCCGGGCTACAGTTGAATGAACAAAGC |  |
| ***Proteolytic activity*** | | |
| Sl3-MMPm-GST-1F | CCCGAATTCGGGAAGTCCACGGTGGCCG | 562 |
| Sl3-MMPm-GST-1R | GGGCTCGAGTTAACTCTCTTGGCTCGGAGTCAAT |  |
| ***qRT-PCR*** | | |
| Sl1-MMP-RT-F | ATCGACGCATCGGAAATTAGA | 102 |
| Sl1-MMP-RT-R | CGCTGTGAAGTAATCCTCTGT |  |
| Sl2-MMP-RT-F | CCCACCATTCAGCATCTCATAA | 83 |
| Sl2-MMP-RT-R | GGCTTTCCAGCGTTCATAGT |  |
| Sl3-MMP-RT-F | CTCTCAAGACCTACCAGCTTAAC | 141 |
| Sl3-MMP-RT-R | GTGGCTTACCGGAGTTCATAG |  |
| Sl4-MMP-RT-F | CTACCGGAGAATCAGTTGACG | 113 |
| Sl4-MMP-RT-R | GCGGATCTATAAGAACCCATCTC |  |
| Sl5-MMP-RT-F | AGAAGCAGTAATGTTCCCTAGTT | 98 |
| Sl5-MMP-RT-R | GGTTTGAACCATAAAGAGCTTGT |  |
| SlActin-RT-F | CCAGGTATTGCTGATAGAATGAG | 113 |
| SlActin-RT-R | GAGCCTCCAATCCAGACAC |  |
| SlPI-I-RT-R | GTTGTACAAATGCCTGTGGTGAC | 135 |
| SlPI-I-RT-R | GGTAAGAGTACATGAAGAGATGC |  |
| SlPI-II-RT-F | CATCTTCTGGATTGCCCA | 106 |
| SlPI-II-RT-R | ACACACAACTTGATGCCCAC |  |
| SlLapA-RT-F | GGGACTAATGATGTTTGGAA | 109 |
| SlLapA-RT-R | GTGGCAATTTTATTTAGGCA |  |
| SlPR1a-RT-F | GGC AGG AAC ACC AAA GAA ACC A | 127 |
| SlPR1a-RT-R | TGG CCT CTG GTC AGG TTT AAA G |  |
| SlPR1b-RT-F | TTTCCCTTTTGATGTTGCT | 96 |
| SlPR1b-RT-R | TGGAAACAAGAAGATGCAGT |  |
| SlRBOH1-RT-1F | GTTGCTGCAGCCATTGTCAC | 130 |
| SlRBOH1-RT-1R | GGCTTGGGCCAAAATCATTC |  |
| SlWfi-RT-F | AGGGAATGATAGAGCGTCG | 143 |
| SlWfi-RT-R | CATCGTCATTGGACTTGGC |  |
| SlCAT1-RT-1F | CCCAGTTAATGCTCCCAAGTGT | 118 |
| SlCAT1-RT-1R | AGGACGACAAGGATCAAACCTC |  |
| SlAPX5-RT-1F | ACTTCACGGAGCTTTTGAGTGG | 141 |
| SlAPX5-RT-1R | CAGCATAGTCAGCAAAGAAGGC |  |
| SlGR1-RT-1F | GATGATGAAATGCGAGCTGTAG | 182 |
| SlGR1-RT-1R | TTTGTGTTAGGGAGACGACCAG |  |
| SlSOD-RT-1F | GGCCAATCTTTGACCCTTTATG | 183 |
| SlSOD-RT-1R | AAGTCCAGGAGCAAGTCCAGTT |  |
| BcActin-RT-F | CGTCACTACCTTCAACTCCATC | 107 |
| BcActin-RT-R | CGGAGATACCTGGGTACATAGT |  |
| NbActin-RT-F | ACCAGATTAATGAGCCCAAGAG | 97 |
| NbActin-RT-R | CCAACAGGGACAGTACCAATAC |  |
| NbPR1-RT-F | CCGTTGAGATGTGGGTCAAT | 100 |
| NbPR1-RT-R | CGCCAAACCACCTGAGTATAG |  |
| NbPR2-RT-F | CAACCCGCCCAAAGATAGTA | 98 |
| NbPR2-RT-R | TGGCTAAGAGTGGAAGGTTATG |  |
| NbPR3-RT-F | GCCAATTCCTTTCCTGGTTT | 94 |
| NbPR3-RT-R | TAGTTTCGTGAGAAGTTTGACCG |  |
| NbPR4-RT-F | GGATGATGTTGACAGCAGAGA | 116 |
| NbPR4-RT-R | GTAGGACACGAGGTAGGTATCA |  |
